# Synergistic Anti-Zika Activity of Repurposed Statin and Clofibrate Combinations: Evidence from Oleic Acid-Mediated Metabolic Complementation

**DOI:** 10.3390/ijms27219637

**Published:** 2026-10-28

**Authors:** Chung-Do Lee, Sang-Ik Park, Gun Lee, Hyeon Jeong Moon, Jaewoo Choi, Jun Bong Lee, Luis Martinez-Sobrido, Fernando Almazán, Eun-Hye Ryu, Yeong-Bin Baek, Jun-Gyu Park

**Affiliations:** 1 Department of Veterinary Pathology, College of Veterinary Medicine and BK21 FOUR Program, Chonnam National University, Gwangju 61186, Republic of Korea; cndehsla@gmail.com (C.-D.L.); sipark@jnu.ac.kr (S.-I.P.); 05311cht@naver.com (J.C.); 2 Animal Medical Institute, Chonnam National University, Gwangju 61186, Republic of Korea; jblee12@jnu.ac.kr; 3 Department of Veterinary Pathology, College of Veterinary Medicine, Chonnam National University, Gwangju 61186, Republic of Korea; udlrjs77@naver.com (G.L.); dals404@naver.com (H.J.M.); 4 Department of Food and Environmental Hygiene, College of Veterinary Medicine, Chonnam National University, Gwangju 61186, Republic of Korea; 5 Texas Biomedical Research Institute, San Antonio, TX 78227, USA; lmartinez@txbiomed.org; 6 Centro Nacional de Biotecnología (CNB-CSIC), Darwin 3, Campus Cantoblanco UAM, 28049 Madrid, Spain; falmazan@cnb.csic.es; 7 Department of Veterinary Zoonotic Diseases, College of Veterinary Medicine, Chonnam National University, Gwangju 61186, Republic of Korea; 1eun00hye4@gmail.com

**Keywords:** Zika virus, dengue virus, drug repurposing, statin, clofibrate, host-directed antiviral, immunometabolism, lipid droplet, metabolic complementation

## Abstract

Zika virus (ZIKV) substantially relies on host lipid metabolism, presenting a prime target for host-directed antivirals. To identify host-directed antiviral interventions while circumventing interferon-mediated transcriptomic masking, we curated a dual-virus (dengue and ZIKV) gene signature to query the Connectivity Map. Top lipid-lowering candidates were validated using a reporter virus (rZIKV-Venus) and an authentic wild-type (WT) ZIKV in Vero E6 cells—a model deliberately selected for its interferon deficiency to isolate pure metabolic responses. Dose–response screening of 67 prioritized compounds identified atorvastatin, rosuvastatin, and clofibrate as potent ZIKV inhibitors. Statin–clofibrate combinations exhibited strong synergy, with fractional inhibitory concentration index (FICI) values of 0.203 (atorvastatin–clofibrate) and 0.117 (rosuvastatin–clofibrate) for the 4:1 clofibrate-to-statin ratio and an optimized selectivity index. Mechanistically, exogenous oleic acid supplementation induced significant metabolic complementation, significantly restoring both host lipid droplet (LD) abundance and ZIKV replication readouts suppressed by the dual treatment. Relative LD intensity strongly correlated with viral intensity in both models (Pearson *r* = 0.951 and 0.956, respectively; both *p* < 0.0001). These findings demonstrate that concurrent pharmacological modulation of host lipid metabolism exerts synergistic anti-ZIKV effects, highlighting highly curated transcriptomic repurposing as a mechanistic prioritization strategy for host-directed antiviral discovery.

## 1. Introduction

Zika virus (ZIKV) is an enveloped, positive-sense RNA arbovirus belonging to the family *Flaviviridae* and is transmitted primarily by *Aedes* mosquitoes, especially *Aedes aegypti* and *Aedes albopictus* [1,2]. Although ZIKV infection is frequently asymptomatic or associated with mild febrile illness, the large outbreaks in the Pacific and the Americas revealed its capacity to cause severe neurological and congenital complications [2,3,4,5]. ZIKV infection has been associated with Guillain–Barré syndrome in adults and with congenital abnormalities, including microcephaly, following infection during pregnancy [3,4,5]. During the 2015–2016 epidemic in the Americas, autochthonous ZIKV transmission was reported across many countries and territories, establishing ZIKV as a major emerging public health concern [6]. Although global ZIKV activity declined after the epidemic, low-level transmission and sporadic outbreaks continue to occur in endemic regions, and the risk of re-emergence of ZIKV remains [7,8,9]. Despite the public health burden, no licensed prophylactic vaccines or specific therapeutic antivirals are currently available for the prevention and treatment, respectively, of ZIKV [8].

Traditional antiviral development targeting specific viral enzymes is frequently hindered by the rapid emergence of escape mutations. Consequently, identifying therapies that modulate host–virus interactions—known as host-directed interventions—has become a central paradigm in modern antiviral research [10]. Viral pathogenesis emerges at the complex intersection of viral replicative programs and host cellular defenses. Specifically, flaviviruses, including ZIKV, are structurally and functionally tethered to host lipid metabolism. Upon cellular entry, ZIKV extensively remodels the host endoplasmic reticulum (ER) and associated endomembrane systems to establish intricate replication compartments, a process intrinsically reliant on de novo lipid biosynthesis, cholesterol trafficking, and the active mobilization of intracellular lipid droplets (LDs) [11,12]. Recent studies have highlighted that ZIKV actively hijacks host LD biogenesis and modulates lipophagy pathways to fuel energy production, support viral RNA replication, and orchestrate the assembly of mature virions [13]. Because this metabolic hijacking and lipid-mediated signaling are essential prerequisites for the viral life cycle, targeting host immunometabolism represents a highly promising, host-directed antiviral strategy.

Drug repurposing, also known as drug repositioning, is a strategy that identifies new therapeutic uses for existing drugs [14]. This approach offers several advantages over de novo drug development, including shorter development time, lower costs, and established safety profiles [14]. Drug repurposing has been successfully applied to identify antiviral candidates against various emerging viruses, including ZIKV [15]. In parallel with conventional phenotypic screening, transcriptomic signature-based drug repurposing provides a complementary systems-biology strategy for identifying compounds that may reverse infection-associated host transcriptional states using the Connectivity Map (CMap) platform [16,17]. However, the direct application of raw viral infection transcriptomes to CMap frequently encounters a critical analytical bias. Because host responses to acute viral infections are predominantly dominated by massive type I interferon (IFN) and inflammatory cascades, computational algorithms often exhibit a pervasive “transcriptomic masking effect.” Consequently, these algorithms may falsely prioritize broad immunosuppressants (e.g., glucocorticoids) that merely blunt inflammation, rather than identifying true antiviral compounds targeting the specific metabolic dependencies of the virus [18]. To overcome this limitation and isolate the pathways associated with viral metabolic manipulation, we employed a highly curated dual-virus transcriptomic approach. By integrating overlapping signatures from both ZIKV and the closely related dengue virus (DENV)—while explicitly filtering out dominant IFN-stimulated genes—we aimed to extract a conserved host metabolic dependency signature applicable for flavivirus-directed in silico prioritization.

Statins are a class of drugs that inhibit 3-hydroxy-3-methylglutaryl-coenzyme A (HMG-CoA) reductase, the rate-limiting enzyme in the mevalonate pathway, thereby reducing cholesterol biosynthesis and altering downstream lipid-associated cellular processes [19]. Fibrates, including clofibrate, regulate lipid and lipoprotein metabolism at least partly through peroxisome proliferator-activated receptor alpha (PPARα)-dependent transcriptional mechanisms [20]. Both statins and fibrates have been investigated for their individual antiviral properties against various viruses, including flaviviruses [21,22]. However, the potential synergistic antiviral effects of concurrently targeting distinct lipid metabolic nodes—such as combining statin-mediated cholesterol depletion with fibrate-mediated fatty acid regulation—against ZIKV pathogenesis have not been systematically elucidated.

In this study, to align with the need for host-directed interventions, we explored a transcriptomic signature-based drug repurposing approach to identify candidate therapeutics against ZIKV infection. We integrated publicly available ZIKV and DENV infection transcriptomic data with the Connectivity Map Linked User Environment (CLUE) database to prioritize approved drugs for in vitro validation. We identified three lipid-lowering drugs (atorvastatin, rosuvastatin, and clofibrate) that exhibited antiviral activity against ZIKV and further evaluated the synergistic antiviral effects of statin–clofibrate combinations.

To mechanistically investigate whether this antiviral synergy is driven by the disruption of viral-associated lipid reserves, we performed oleic acid (OA)-mediated metabolic complementation assays. By quantitatively tracking the dynamic interplay between host LDs and concurrent viral replication readouts across both reporter and authentic wild-type (WT) ZIKV models, this study provides objective evidence supporting host lipid metabolism as a druggable axis for ZIKV inhibition.

## 2. Results

### 2.1. Transcriptome-Guided Prioritization Identified 67 Candidate Compounds for Flaviviral Screening

**Figure 1:**
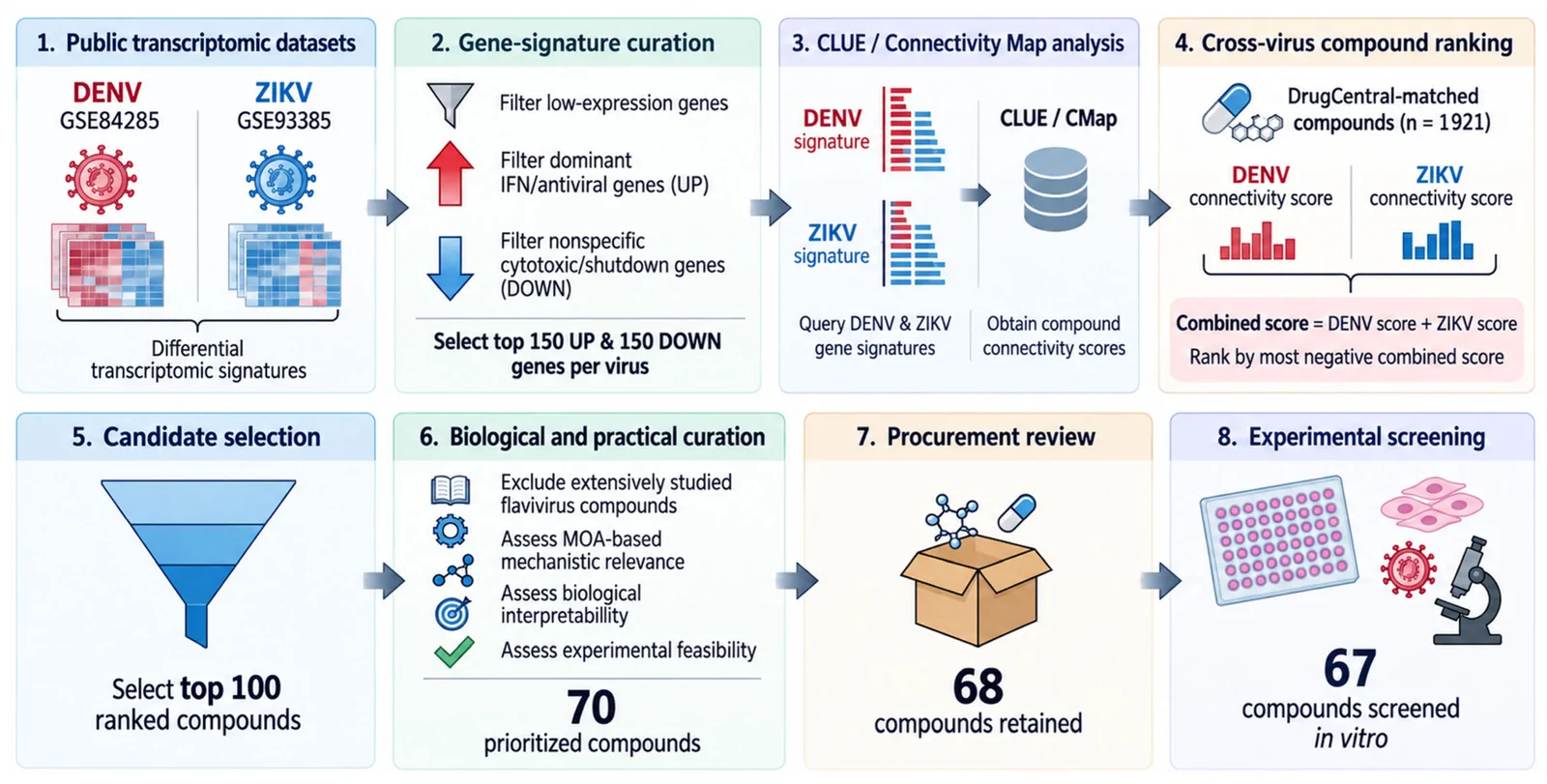
Transcriptome-guided compound prioritization workflow for anti-ZIKV screening. Publicly available dengue virus (DENV; GSE84285) and Zika virus (ZIKV; GSE93385) transcriptomic datasets were integrated. Following the rigorous filtration of low-expression genes, dominant interferon (IFN)-stimulated genes (to prevent transcriptomic masking bias), and non-specific cytotoxicity genes, dual-virus expression signatures were generated. These signatures were queried against the Connectivity Map Linked User Environment (CLUE)/Connectivity Map (CMap) platform using specific parameters detailed in Supplementary Table S2. DrugCentral-matched compounds were ranked by a combined negative connectivity score. The top 100 candidates underwent objective biological curation based on mechanism-of-action diversity and translational potential, resulting in 67 structurally diverse compounds selected for in vitro phenotypic screening.

To rationally identify approved-drug-derived candidates with anti-ZIKV potential against flavivirus infections, we executed a transcriptome-guided drug repurposing workflow. Rather than relying solely on a single virus model, which is highly susceptible to strain- or cell-line-specific artifacts, we integrated both DENV and ZIKV infection transcriptomic datasets (GSE84285 and GSE93385) to extract conserved host metabolic dependencies. Crucially, to prevent the computational algorithm from prioritizing generalized immunosuppressants—a confounding phenomenon known as the “transcriptomic masking effect”—dominant innate antiviral and IFN-stimulated genes were filtered from the query signatures prior to analysis (Figure 1).

Following in silico querying against the CLUE CMap and matching with the DrugCentral database, 1921 compounds were ranked by a combined negative connectivity score, a consensus metric used to mitigate dataset-specific noise. To eliminate redundant pharmacological classes and select candidates with realistic translational potential, the top-ranked in silico candidates underwent objective manual curation based on predefined criteria, prioritizing mechanism-of-action diversity—particularly host lipid modulation—and commercial availability. Ultimately, 67 structurally and pharmacologically diverse candidate compounds were selected for in vitro phenotypic screening (Table 1 and Supplementary Table S1). Notably, cardiovascular and lipid-regulating drugs emerged as a prominent functional cluster within the validation panel, providing a strong computational rationale to investigate host lipid modulation as a therapeutic axis against ZIKV.

### 2.2. Comprehensive Dose–Response Screening Identified Lipid-Lowering Drugs as Potent ZIKV Inhibitors

The antiviral potential of the 67 prioritized candidates was initially evaluated using a recombinant ZIKV expressing Venus fluorescent protein (rZIKV-Venus) reporter assay in Vero E6 cells. To ensure data transparency and directly address the quantitative basis for candidate prioritization, complete dose–response curves were evaluated for all 67 compounds. The resulting half-maximal cytotoxic concentration (CC_50_), half-maximal inhibitory concentration (IC_50_), and selectivity index (SI) values are comprehensively reported in Table 1 (calculated values exceeding the maximum tested concentration of 100 µM were conservatively reported as >100 µM to accurately reflect the assay limits). Acknowledging that reporter fluorescence serves as a proxy for viral protein accumulation and initial genome translation rather than a direct, absolute measure of infectious progeny release, this high-throughput assay was deployed strictly as a primary screen. Furthermore, to exclude solvent-induced experimental bias, all test compounds and vehicle controls were normalized to a final concentration of 1% dimethyl sulfoxide (DMSO).

**Figure 2:**
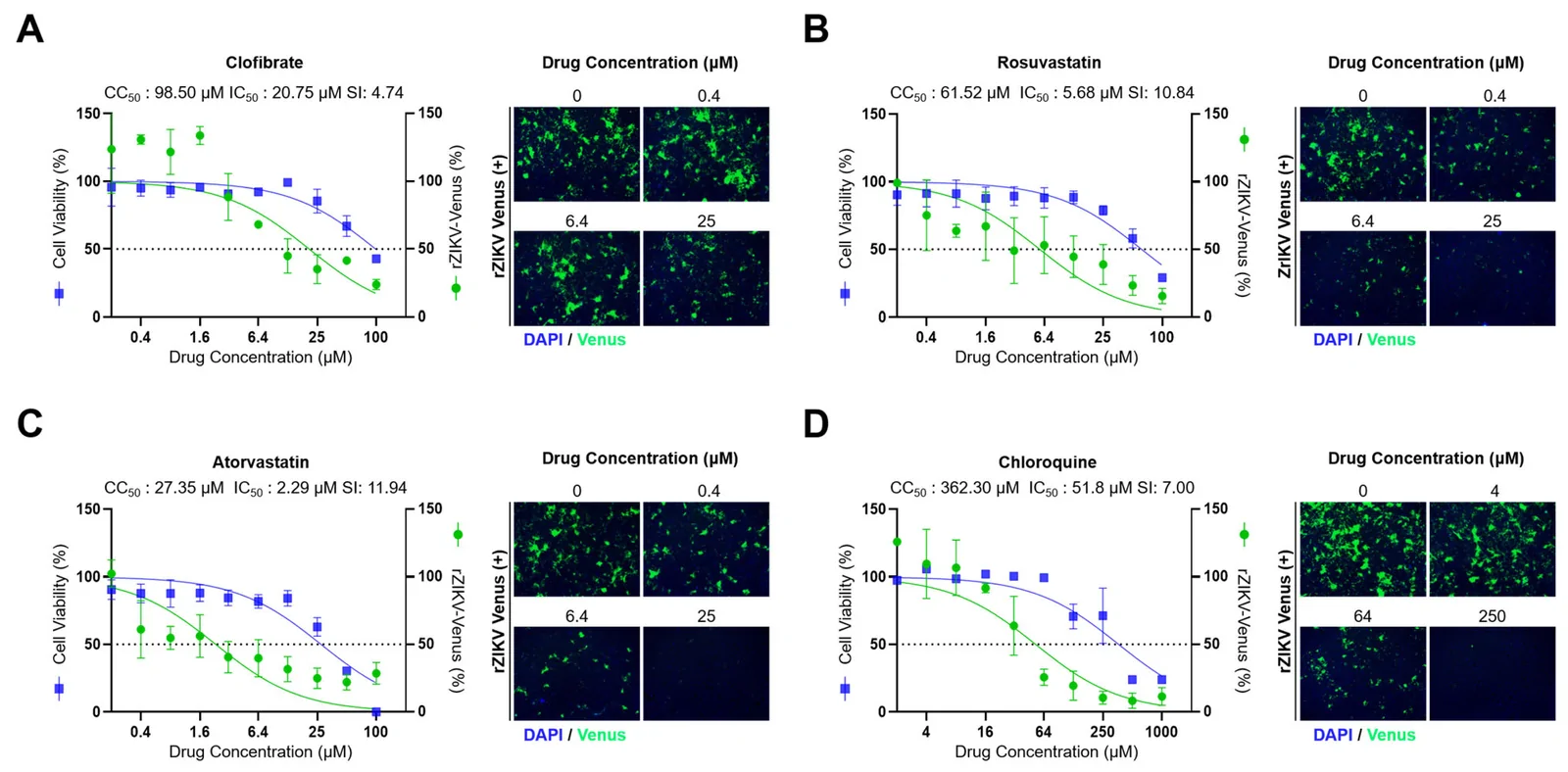
Lipid-lowering drugs suppressed rZIKV-Venus reporter readouts and exhibited favorable cytotoxicity profiles in Vero E6 cells. Dose–response and cytotoxicity profiles of selected candidates. Vero E6 cells were infected with rZIKV-Venus (MOI = 0.025) and continuously exposed to serially diluted compounds for 120 h. Green circles indicate relative Venus fluorescence (proxy for viral translation), and blue squares indicate cell viability (measured via MTT assay), both normalized to vehicle-treated infected controls. Representative fluorescence images correspond to selected drug concentrations. (**A**) Clofibrate. (**B**) Rosuvastatin. (**C**) Atorvastatin. (**D**) Chloroquine (reference compound). Data are presented as the mean ± SD of three independent biological replicates (*n* = 3). Non-linear regression (four-parameter logistic model) was used to extrapolate IC_50_ and CC_50_ values. Abbreviations: MOI, multiplicity of infection; SD, standard deviation; IC_50_, half-maximal inhibitory concentration; CC_50_, half-maximal cytotoxic concentration.

While several candidates from diverse pharmacological classes exhibited measurable antiviral profiles with high selectivity indices (e.g., palonosetron hydrochloride and acetohydroxamic acid), our mechanistic objective was to explicitly interrogate the dependency of ZIKV on host lipid metabolism. Therefore, we focused our downstream validation on the lipid-lowering agents. Three specific lipid-lowering drugs—clofibrate, rosuvastatin, and atorvastatin—exhibited a dose-dependent suppression of rZIKV-Venus reporter expression (Figure 2). To delineate the therapeutic window, metabolic cytotoxicity was determined via the 3-(4,5-dimethylthiazol-2-yl)-2,5-diphenyltetrazolium bromide (MTT) reduction assay. Furthermore, to orthogonally confirm that the metabolic readouts were not confounded by the metabolism-perturbing nature of the drugs, cell number at the working concentrations used in the mechanistic assays was confirmed independently by direct DAPI-positive nuclear counting (Supplementary Figure S2B). This orthogonal check was performed at single working doses only and therefore does not provide an independent CC_50_; all CC_50_ and SI values reported in this study remain MTT-derived. Clofibrate demonstrated an IC_50_ of 20.75 μM and a CC_50_ of 98.50 μM, yielding an SI of 4.74 (Figure 2A). Rosuvastatin exhibited an IC_50_ of 5.68 μM and a CC_50_ of 61.52 μM (SI = 10.83) (Figure 2B), while atorvastatin showed an IC_50_ of 2.29 μM and a CC_50_ of 27.35 μM (SI = 11.94) (Figure 2C). The reference compound chloroquine, evaluated over an extended concentration range to fully capture its upper baseline, suppressed reporter signals with an IC_50_ of 51.8 μM and a CC_50_ of 362.30 μM (SI = 7.00) (Figure 2D).

### 2.3. Statin–Clofibrate Combinations Synergistically Inhibited ZIKV Reporter Translation

**Figure 3:**
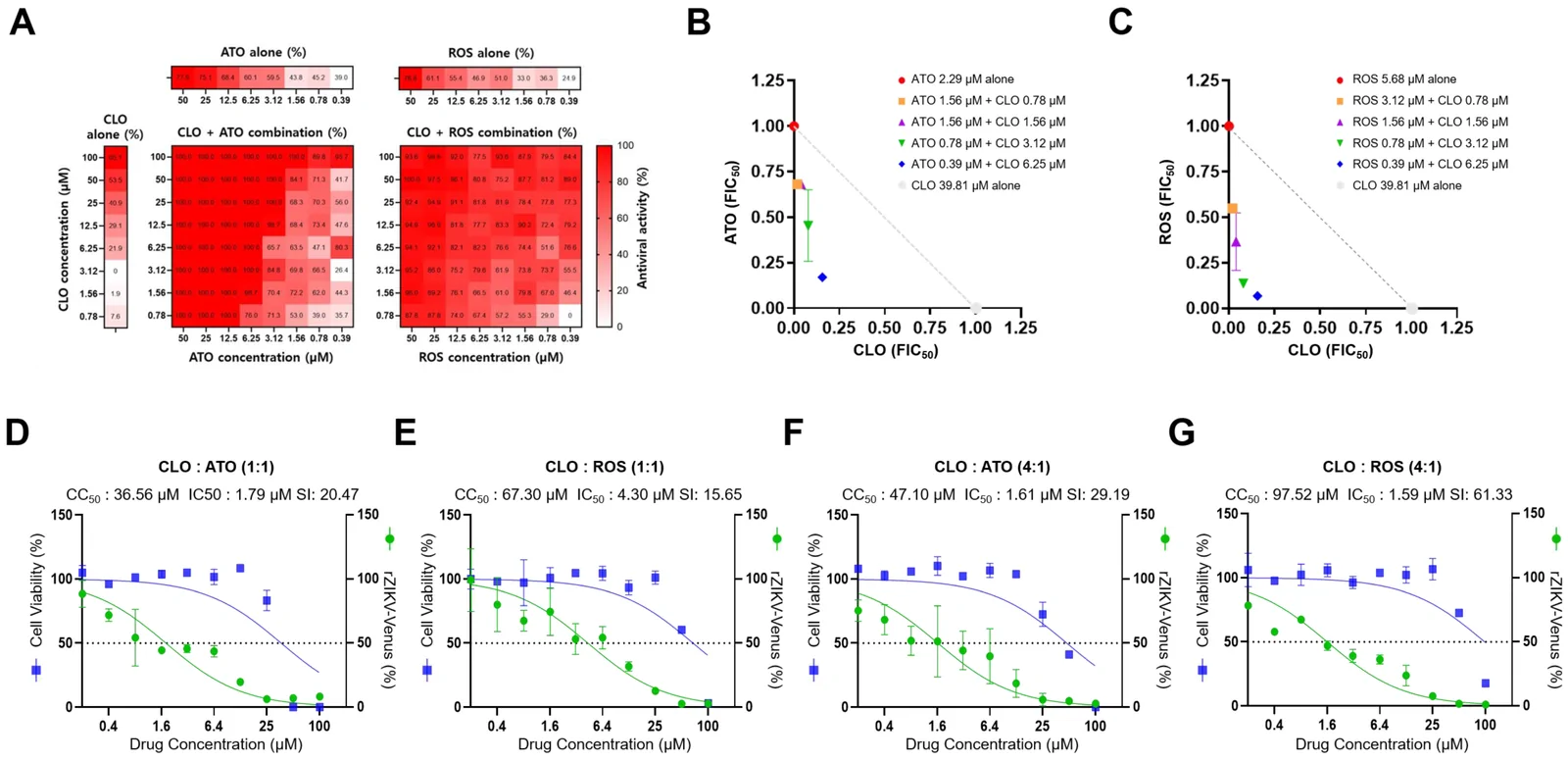
Statin–clofibrate combinations exerted synergistic antiviral activity and expanded the therapeutic window. Drug–drug interactions were quantitatively assessed using the fractional inhibitory concentration index (FICI) via rZIKV-Venus screening. (**A**) Combination matrix heatmaps illustrating the comprehensive dose-dependent suppression of viral activity (%) across varying concentrations of clofibrate (CLO) combined with atorvastatin (ATO) or rosuvastatin (ROS). (**B**,**C**) Isobologram analyses of the (**B**) ATO + CLO and (**C**) ROS + CLO combinations extracted from the matrix data. These plots display the normalized fractional inhibitory concentrations (FICs) of individual agents. All tested fixed-ratio data points localized strictly below the theoretical line of additivity (dotted line). The synergistic points, derived from the 4:1 clofibrate-to-statin ratio, yielded FICI values of 0.203 (ATO) and 0.117 (ROS) satisfying the pharmacological synergy threshold (FICI ≤ 0.5). (**D**–**G**) Representative dose–response and MTT viability curves for fixed-ratio combinations. The optimized 4:1 CLO:ROS ratio (**G**) achieved maximal synergistic efficacy (IC_50_ = 1.59 μM) and an optimized selectivity index (SI = 61.33). Data represent the mean ± SD (*n* = 3).

To investigate whether concurrent targeting of distinct nodes within host lipid metabolism could potentiate antiviral efficacy, combination drug assays were performed using fixed concentration ratios. We first evaluated the interaction landscape across varying concentration grids. Combination matrix analysis visually demonstrated an enhanced and broadened dose-dependent suppression of rZIKV-Venus by the statin-clofibrate dual treatments compared to their respective monotherapies (Figure 3A).

To mathematically quantify this observed enhancement, the fractional inhibitory concentration index (FICI) was calculated by extracting the specific concentrations of each individual agent within the mixture required to achieve 50% inhibition. On the resulting isobolograms, all tested fixed-ratio data points were explicitly located below the theoretical line of additivity. The synergistic points, derived from the 4:1 clofibrate-to-statin ratio, yielded FICI values of 0.203 for atorvastatin-clofibrate (Figure 3B) and 0.117 for rosuvastatin-clofibrate (Figure 3C). The calculated FICI values for all tested ratios (4:1, 2:1, 1:1, 1:2, and 1:4, expressed as clofibrate-to-statin), together with the replicate-level values and 95% confidence intervals, are comprehensively reported in Supplementary Table S3. Because both calculated indices fall below the established pharmacological synergy threshold (FICI ≤ 0.5), these spatial and mathematical data indicate a synergistic antiviral interaction. To orthogonally validate this synergistic interaction, we performed response-surface analyses using the Zero Interaction Potency (ZIP) and Bliss reference models via SynergyFinder. The resulting 3D synergy landscapes corroborated the robust synergistic suppression of ZIKV across the concentration grids (Supplementary Figure S1).

Crucially, the statin–clofibrate combinations significantly potentiated antiviral activity without causing proportional increases in host cytotoxicity. Among the evaluated regimens, the 4:1 fixed ratio of clofibrate to rosuvastatin exhibited an optimized profile, achieving an IC_50_ of 1.59 μM and expanding the SI to 61.33 (Figure 3D–G); this selectivity index, like all others reported in this study, is MTT-derived. Given this optimal balance of synergistic efficacy and improved safety margin, the 4:1 clofibrate-to-rosuvastatin combination was prioritized for all subsequent mechanistic complementation assays.

### 2.4. OA-Mediated Metabolic Complementation Confirms the LD Dependency of ZIKV Translation and Progeny Production

To mechanistically determine whether the synergistic antiviral effect of the rosuvastatin–clofibrate combination was fundamentally driven by the depletion of host lipid reserves, we investigated whether exogenous lipid supplementation could complement the drug-induced metabolic deficit. Vero E6 cells infected with rZIKV-Venus were treated with the optimized 4:1 synergistic drug combination (rosuvastatin and clofibrate), OA alone, or the triple combination (all treatments added simultaneously at 1 h post-infection).

**Figure 4:**
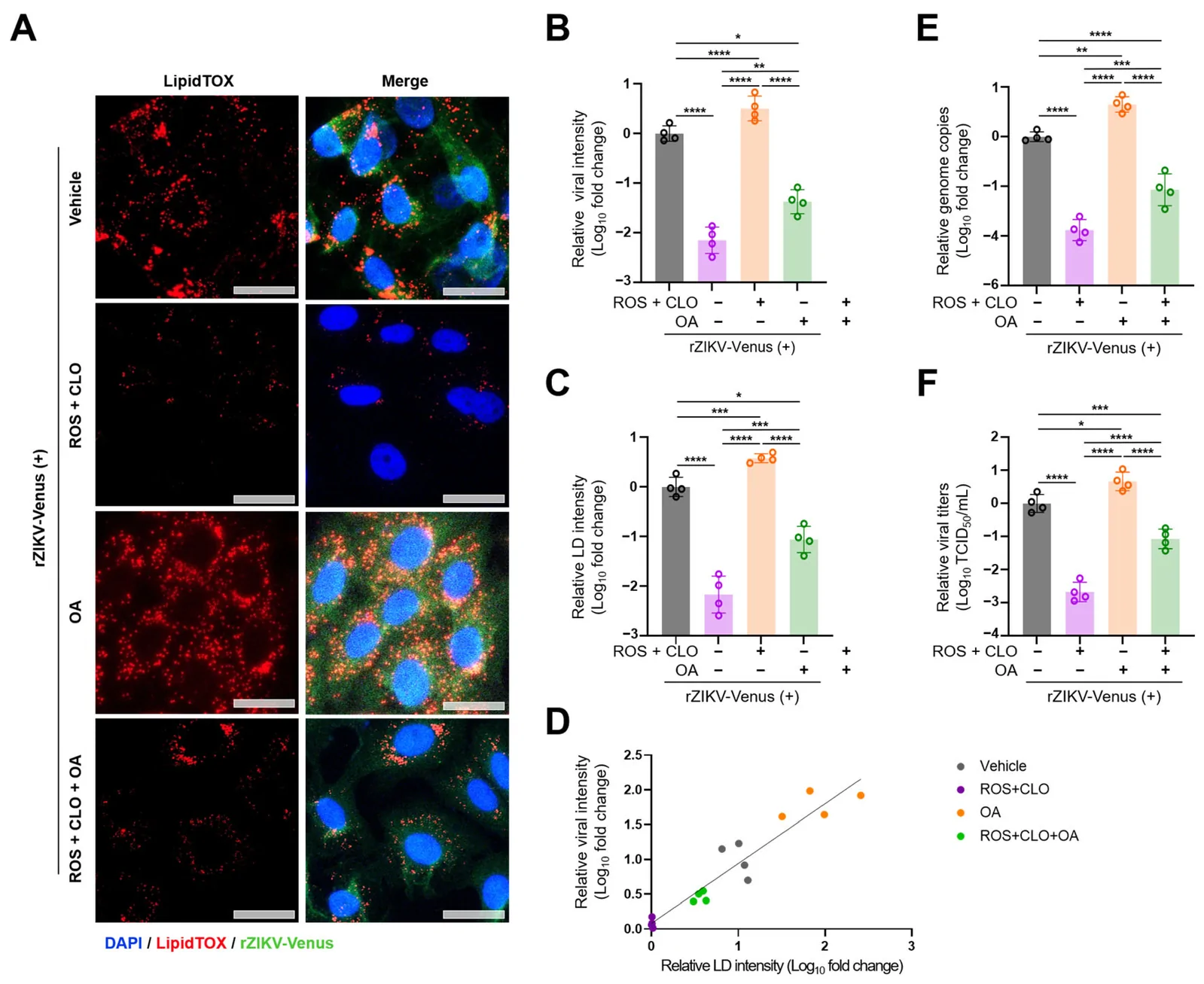
Oleic acid (OA)-mediated metabolic complementation confirms the lipid droplet (LD) dependency of rZIKV-Venus. Vero E6 cells were infected with rZIKV-Venus (MOI = 0.025) and treated with 1% DMSO vehicle, rosuvastatin + clofibrate (ROS + CLO; 0.78 μM + 3.12 μM), OA alone (100 μM), or the triple combination (ROS + CLO + OA) for 120 h. (**A**) Representative confocal images displaying rZIKV-Venus (green), LipidTOX-stained neutral LDs (red), and DAPI-stained nuclei (blue). Scale bars = 30 μm. (**B**,**C**) Quantitative image analysis of relative rZIKV-Venus intensity (**B**) and relative LipidTOX intensity (**C**), expressed as log_10_ fold changes relative to the vehicle control. (**D**) Pearson correlation analysis and simple linear regression between log_10_-transformed LD intensity and viral reporter intensity (pooled Pearson r = 0.951, *p* < 0.0001; dotted lines represent the 95% confidence bands). Each point represents one independent biological replicate (*n* = 4 per treatment group; 16 points in total). The coefficient is computed across all four treatment groups pooled; because the groups were designed to differ in both lipid droplet abundance and viral signal and separate along both axes, it reflects between-group separation as well as any within-group association and should not be read as evidence of a graded within-group relationship. (**E**) RT-qPCR quantification of relative intracellular viral RNA genome copies from cell pellets, expressed as log_10_ fold changes and normalized to endogenous β-actin. (**F**) Infectious viral titers (log_10_ TCID_50_/mL) determined following three freeze–thaw cycles to measure total infectious progeny. Data are presented as the mean ± SD (*n* = 4 biological replicates). Statistical significance was determined using one-way ANOVA followed by Tukey’s post hoc test (* *p* < 0.05, ** *p* < 0.01, *** *p* < 0.001, **** *p* < 0.0001). Abbreviations: RT-qPCR, reverse transcription quantitative PCR; TCID_50_, 50% tissue culture infectious dose; ANOVA, analysis of variance.

Confocal microscopy provided initial qualitative evidence of the dynamic interplay between host neutral LDs and viral reporter expression (Figure 4A). To establish a rigorous quantitative framework, we extracted single-cell-level fluorescence intensities and calculated the log_10_-transformed fold changes relative to the vehicle-treated control. The combination treatment markedly suppressed rZIKV-Venus reporter accumulation (Figure 4B) with a concurrent, substantial depletion of host LDs (Figure 4C). Conversely, OA monotherapy significantly expanded the host LD pool and proportionally enhanced viral reporter signals. Rather than contradicting the therapeutic premise, this pro-viral effect of exogenous lipids provides mechanistic evidence that intracellular ZIKV translation is constrained by host LD availability. However, it is noteworthy that OA primarily drives diacylglycerol acyltransferase (DGAT)-dependent triacylglycerol storage, which may not fully replenish the specific cholesteryl-ester pools depleted by statins. This is one possible explanation for the partial rather than complete complementation observed, but it was not tested here and remains hypothetical. Consequently, when OA was supplemented to the statin–clofibrate treatment regimen, it partially compensated for the drug-induced lipid deficit, leading to a statistically significant metabolic complementation that restored both LD abundance and rZIKV-Venus expression (Figure 4B,C). Furthermore, we confirmed via MTT assay that OA supplementation, either alone or in combination with the drugs, did not induce significant cytotoxicity, ensuring that differences in cell viability did not confound the fluorescence intensity normalizations (Supplementary Figure S2).

To mathematically evaluate this observed dependency, we performed a Pearson correlation analysis. The log_10_-transformed relative LD intensity exhibited a strong, significant positive linear correlation with viral reporter intensity (Pearson *r* = 0.951, *p* < 0.0001) (Figure 4D). Moving from single-cell viral translation to bulk genome replication, reverse transcription quantitative PCR (RT-qPCR) analysis of intracellular RNA similarly demonstrated that OA supplementation partially rescued the suppressive effect of the drugs on viral genome replication (Figure 4E). Finally, to definitively evaluate the end-stage production of functional infectious progeny, a modified 50% tissue culture infectious dose (TCID_50_) assay using repeated freeze–thaw cycles was performed to encompass both intracellularly retained and extracellularly released virions. The combination treatment markedly reduced infectious viral titers, whereas OA supplementation triggered a significant, albeit partial, metabolic complementation of progeny production (Figure 4F).

### 2.5. Phenotypic Validation Against Authentic WT ZIKV (PRVABC59)

Because reporter viruses may exhibit altered replication kinetics or fluorescent artifacts, we validated the lipid-dependent antiviral phenotype using an authentic, replication-competent clinical isolate, WT ZIKV (PRVABC59). The analytical workflow mirrored the reporter model, using the pan-flavivirus 4G2 monoclonal antibody to specifically detect WT ZIKV envelope protein accumulation.

**Figure 5:**
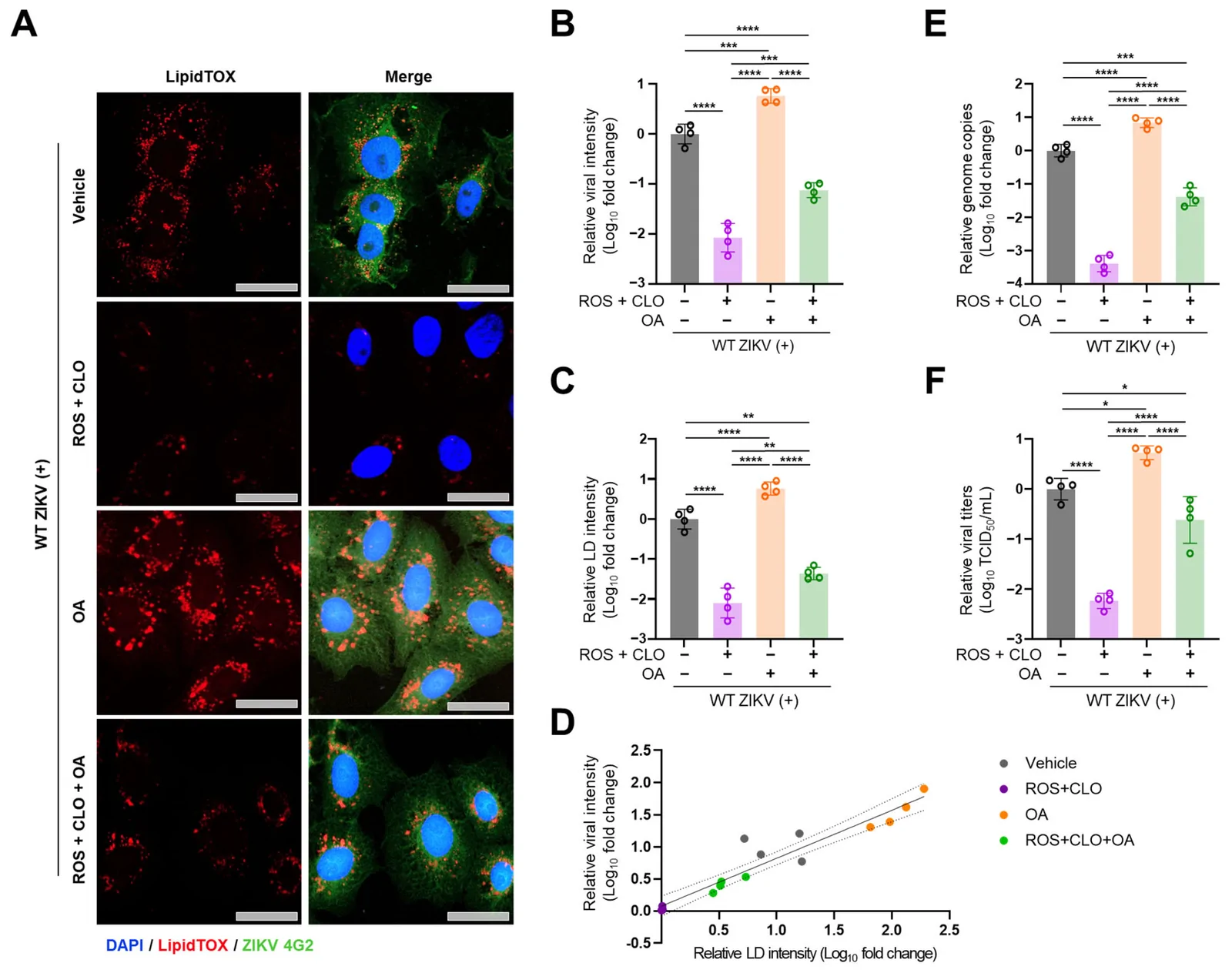
Phenotypic validation of lipid-dependent viral suppression against authentic wild-type (WT) ZIKV (PRVABC59). The metabolic complementation assay was replicated using the replication-competent WT ZIKV strain (MOI = 0.025). (**A**) Representative confocal images. Viral envelope proteins were visualized using the pan-flavivirus 4G2 monoclonal antibody (green), alongside LipidTOX (red) and DAPI (blue). Scale bars = 30 μm. (**B**,**C**) Quantitative image analysis of relative 4G2 intensity (**B**) and relative LipidTOX intensity (**C**), expressed as log_10_ fold changes relative to the vehicle control. (**D**) Pearson correlation analysis and simple linear regression between log_10_-transformed LD intensity and viral reporter intensity (pooled Pearson r = 0.956, *p* < 0.0001; dotted lines represent the 95% confidence bands). Each point represents one independent biological replicate (*n* = 4 per treatment group; 16 points in total). The coefficient is computed across all four treatment groups pooled; because the groups were designed to differ in both lipid droplet abundance and viral signal and separate along both axes, it reflects between-group separation as well as any within-group association and should not be read as evidence of a graded within-group relationship. (**E**) RT-qPCR quantification of relative intracellular WT ZIKV RNA genome copies from cell pellets, expressed as log_10_ fold changes and normalized to endogenous β-actin. (**F**) Infectious viral titers (log_10_ TCID_50_/mL) determined from cell lysates following freeze–thaw cycles. Data are presented as the mean ± SD (*n* = 4 biological replicates). Statistical significance was determined using one-way ANOVA followed by Tukey’s post hoc test (* *p* < 0.05, ** *p* < 0.01, *** *p* < 0.001, **** *p* < 0.0001).

Consistent with the reporter model, confocal imaging demonstrated that the rosuvastatin–clofibrate combination markedly depleted cellular LDs and strongly suppressed WT ZIKV envelope protein accumulation (Figure 5A). Quantitative single-cell image analysis (log_10_ fold change) confirmed that OA supplementation induced metabolic complementation, significantly restoring both WT viral antigen expression (Figure 5B) and LD intensity (Figure 5C) in the drug-treated cells. Lipid droplet abundance and WT ZIKV 4G2 envelope accumulation moved together across the four treatment conditions (pooled Pearson r = 0.956, *p* < 0.0001) (Figure 5D).

Downstream virological assessments corroborated the imaging results. Intracellular genome quantification revealed a strong suppression of WT ZIKV RNA replication by the dual drug treatment, which was significantly reversed by OA addition (Figure 5E). Furthermore, TCID_50_ infectious progeny titration confirmed that the statin–clofibrate synergistic mechanism impairs ZIKV replication through significant host lipid metabolic disruption, culminating in a significant reduction in infectious virion release that is partially complementable by exogenous fatty acids (Figure 5F).

## 3. Discussion

In this study, aligning with the urgent need for host-directed anti-ZIKV interventions, we used a transcriptomic signature-based drug repurposing strategy to identify approved-drug-derived candidates with potential anti-ZIKV activity and validated selected compounds using both an rZIKV-Venus reporter infection system and an authentic WT ZIKV strain. From 67 candidate compounds selected through connectivity-based prioritization and manual curation, three lipid-lowering drugs (atorvastatin, rosuvastatin, and clofibrate) reduced ZIKV translation and replication readouts in Vero E6 cells. Moreover, statin–clofibrate combinations showed synergistic antiviral activity, as indicated by FICI values of 0.203 and 0.117, and markedly improved the therapeutic safety margin (SI = 61.33). Importantly, OA-mediated metabolic complementation experiments support a lipid-dependent mechanism, as exogenous OA supplementation partially restored LD abundance and concurrent ZIKV-associated readouts suppressed by rosuvastatin and clofibrate. These findings suggest that targeting host lipid metabolism represents a promising strategy for developing host-directed antiviral therapies against ZIKV and potentially other flavivirus infections.

A methodological strength of our in silico approach was the integration of both DENV and ZIKV transcriptomes, coupled with the exclusion of dominant IFN-stimulated genes. Direct application of raw viral infection signatures to the CMap platform frequently induces a “transcriptomic masking effect,” wherein algorithms falsely prioritize generalized immunosuppressants (e.g., glucocorticoids) due to the innate inflammatory noise [18]. By uncoupling this generic immune response, we successfully isolated a conserved, pan-flaviviral host metabolic dependency signature. Furthermore, while initial in vitro screening identified several compounds with higher individual selectivity indices (e.g., palonosetron hydrochloride, SI > 12.64), we prioritized statins and fibrates. Our primary objective was not merely to identify non-specific inhibitors, but to mechanistically interrogate the dependency of ZIKV on host lipid metabolism—a core driver of viral pathogenesis.

Among the selected compounds, atorvastatin, rosuvastatin, and clofibrate showed measurable inhibition of ZIKV replication parameters. Cholesterol depletion disrupts lipid raft organization in cellular membranes, which are cholesterol- and sphingolipid-enriched microdomains that serve as platforms for viral entry, assembly, and budding [23]. Flaviviruses, including ZIKV, extensively remodel host cell membranes to create replication compartments, and this process is highly dependent on cholesterol availability and lipid metabolism [11,12,13,22,24,25,26,27,28,29]. Previous reports also showed that lipidomic and transcriptomic remodeling during ZIKV infection can involve sphingolipid networks, sterol regulatory element-binding protein 2 (SREBP2)-dependent lipid gene expression, glycosphingolipids, and LD-associated processes [13,25,26,27,28]. Among the compounds used in this study, statins (atorvastatin and rosuvastatin) are known to inhibit HMG-CoA reductase, the rate-limiting enzyme in the mevalonate pathway, thereby depleting not only cholesterol but also isoprenoid intermediates such as geranylgeranyl pyrophosphate (GGPP) and farnesyl pyrophosphate (FPP) [19,21,30]. Furthermore, GGPP and FPP are essential for protein prenylation, a post-translational modification that anchors small GTPases such as Rho, Rac, and Rab to cellular membranes [31,32]. These prenylated GTPases regulate membrane trafficking, cytoskeletal dynamics, and signal transduction pathways that are exploited by viruses for replication complex assembly and virion transport [31]. Additionally, statins interfere with viral glycoprotein processing and incorporation into virions, as demonstrated for Ebola virus (EBOV), further compromising viral infectivity [33]. Collectively, these pleiotropic effects, including cholesterol depletion, lipid raft disruption, and impaired protein prenylation, converge to inhibit multiple stages of the ZIKV replicative cycle [21,30,32,34].

The strong synergy observed with statin–clofibrate combinations fundamentally arises from the concurrent modulation of distinct pathways within of host lipid metabolism. While statins primarily disrupt the mevalonate pathway and deplete cellular cholesterol pools, clofibrate, a fibrate-class lipid-lowering drug, modulates fatty acid and triglyceride metabolism through PPARα-dependent transcriptional regulation [20,35]. Clofibrate was selected objectively based on its high in silico connectivity ranking to serve as a pharmacological probe. We acknowledge that in vivo, it is rapidly hydrolyzed to its active metabolite, clofibric acid, and has limited contemporary clinical use due to safety profiles. PPARα is a nuclear receptor that, upon activation by fibrates, upregulates genes involved in fatty acid β-oxidation and downregulates genes involved in lipid biosynthesis [35]. LDs are dynamic organelles that play critical roles in ZIKV replication by serving as platforms for viral protein localization, replication complex assembly, and virion morphogenesis [13,22]. ZIKV infection has been shown to increase LD abundance and to trigger autophagy-mediated lipophagy to mobilize stored lipids for membrane remodeling and energy production [22,25]. By simultaneously disrupting cholesterol synthesis (via statins) and fatty acid/triglyceride homeostasis (via clofibrate), statin–clofibrate combinations may impair the lipid-rich replication compartments required for efficient ZIKV replication. Consistent with this hypothesis, statin–clofibrate two-drug combinations yielded FICI values below 0.5 and exhibited improved antiviral selectivity relative to single-agent treatment. Because ZIKV replication depends on multiple lipid-associated processes, concurrent modulation of distinct lipid metabolic pathways may suppress viral replication more effectively than targeting a single pathway alone. Furthermore, fibrate-mediated enhancement of fatty acid β-oxidation may reduce the availability of fatty acids required for phospholipid synthesis and membrane expansion, further limiting the virus’s capacity to remodel host membranes [35]. Collectively, these complementary effects on host lipid metabolism likely contribute to the synergistic antiviral activity and enhanced selectivity observed with statin–clofibrate combinations. However, the precise molecular basis of this interaction remains to be determined and will require further validation through targeted lipidomic, genetic, and metabolic complementation-based studies.

A key aspect of our mechanistic investigation is the OA-mediated metabolic complementation assay. OA is a monounsaturated fatty acid that serves as a key substrate for triglyceride synthesis and LD biogenesis [13,22]. Upon cellular uptake, OA is esterified by diacylglycerol acyltransferases (DGAT1 and DGAT2) to form triglycerides, which are then packaged into nascent LDs in the ER [36]. Notably, our results demonstrated that exogenous OA monotherapy amplified ZIKV replication readouts. Rather than constituting a confounding artifact, this pro-viral effect provides biological evidence that intracellular ZIKV translation and virion assembly are constrained by host LD availability. In this study, when OA was concurrently co-administered to cells treated with the rosuvastatin–clofibrate combination, it partially compensated for the pharmacological blockade, partially restoring both LD intensity and ZIKV signal intensity across both reporter and authentic WT strains. Lipid droplet abundance and viral readouts moved together across the four treatment conditions in both models (pooled Pearson *r* = 0.951 and 0.956 for the reporter and WT models, respectively; both *p* < 0.0001), which is consistent with host LD availability contributing to ZIKV replication efficiency. Because these coefficients are computed across treatment groups that differ by design, they describe concordance between conditions rather than a graded within-group relationship. These observations are consistent with previous reports linking LDs and fatty acid metabolism to ZIKV replication [13,22,36]. However, it is important to note that OA supplementation did not fully restore viral replication, suggesting that the antiviral effects of statins and clofibrate are not solely attributable to LD depletion. As OA primarily drives DGAT-dependent triacylglycerol (TAG) storage, it may not completely restore the sterol O-acyltransferase (SOAT)-dependent cholesteryl-ester (CE) pools depleted by the statin–clofibrate combination. This lipid-class-specific difference may plausibly explain why OA supplementation resulted in partial, rather than complete, restoration of viral replication [36]. We emphasise that this remains a hypothesis: lipid-class profiling and SOAT/DGAT perturbation were not performed, and the relative contributions of the cholesteryl-ester and triacylglycerol pools to ZIKV replication in this model therefore remain undetermined. This, alongside additional lipid-independent mechanisms likely contribute, including impaired protein prenylation (due to GGPP/FPP depletion by statins), altered membrane composition and fluidity (due to cholesterol depletion), modulation of autophagy and lipophagy pathways, and potential immunomodulatory effects of these drugs [22,25,31,32]. Therefore, without specific pathway-rescue experiments (e.g., direct mevalonate or geranylgeranyl pyrophosphate add-back), the observed antiviral effects cannot be exclusively attributed solely to HMG-CoA reductase inhibition.

Table 2 places the concentrations used here in a clinical context. The in vitro IC_50_ values exceed the unbound maximal plasma concentrations achievable at the highest approved doses by approximately three orders of magnitude for both statins. Exposure at the tissues relevant to ZIKV pathogenesis is unlikely to exceed plasma exposure: rosuvastatin is hydrophilic and penetrates the central nervous system poorly, and atorvastatin is subject to efflux at the blood–brain and placental barriers. In the absence of direct measurements of unbound statin concentrations in brain or placental tissue, we therefore regard sustained antivirally active exposure at these sites as implausible at approved doses. These compounds were used here as in vitro pharmacological probes to test a metabolic dependency, and the present data do not support the repurposing of statins at clinical doses as anti-ZIKV therapy; the translational value of this work lies in identifying the dual lipid-node dependency itself as a target.

This study has some limitations that highlight avenues for future research. First, the antiviral validation was performed primarily in Vero E6 cells. While the lack of physiological relevance to human neural or placental tissues is acknowledged, the deliberate selection of the Vero E6 line was a strict methodological necessity for our mechanistic proof-of-concept. Because Vero E6 cells inherently harbor genomic deletions in the type I IFN gene cluster, they provide a highly controlled environment [40]. This unique characteristic allowed us to isolate and evaluate the direct consequences of pharmacological lipid depletion on ZIKV replication, minimizing the confounding effects of the parallel activation of host innate immune responses. As this study was framed strictly as a mechanistic proof-of-concept, future translational studies must validate these findings in human primary neural progenitor cells and placental trophoblasts. Second, while we addressed the limitations of reporter-based screens by integrating rigorous RT-qPCR and freeze–thaw TCID_50_ titration of authentic WT ZIKV—confirming significant reductions in functional infectious progeny—the tested compounds were evaluated in vitro, and for the reasons set out above, they must be regarded as pharmacological probes rather than as candidates for clinical translation. Third, the mechanism underlying statin–clofibrate synergy remains unresolved. Further work using comprehensive lipid-class profiling (lipidomics), pathway-specific metabolic complementation experiments (e.g., mevalonate or GGPP/FPP add-back assays), and genetic perturbation of lipid metabolic regulators (e.g., SOAT/DGAT modulation) will be needed to fully define the relevant host pathways. Fourth, clofibrate is a prodrug that is rapidly hydrolysed by plasma and tissue carboxylesterases to its active metabolite, clofibric acid. We did not verify whether this conversion occurs in Vero E6 cells, and neither species was quantified in the culture medium or in cell lysates. The active species responsible for the antiviral activity reported here is therefore undefined: it may be the intact prodrug, the hydrolysed acid, or both. For this reason clofibrate is deliberately excluded from the exposure comparison in Table 2, because the available clinical pharmacokinetic data describe circulating clofibric acid and cannot be compared with the in vitro potency of the applied prodrug without knowing the extent of intracellular conversion in this model. Quantification of both species by LC–MS/MS in exposed cultures, together with a side-by-side comparison of clofibrate and clofibric acid potency, is required before the clofibrate arm of this combination can be interpreted in exposure terms.

## 4. Materials and Methods

### 4.1. Transcriptomic Datasets and Generation of Dual-Virus Infection Signatures

To capture conserved flavivirus-induced host transcriptional states, publicly available RNA-seq datasets for DENV and ZIKV infections were retrieved from the NCBI Gene Expression Omnibus (GEO) under accession numbers GSE84285 and GSE93385, respectively. For the DENV dataset, gene-level read counts were normalized using the median-of-ratios method in the DESeq2 R package (version 1.38.3) [41,42]. For the ZIKV dataset, pre-processed differential expression data were used. To enhance the mechanistic specificity of the signatures, raw gene lists were curated. Low-expression genes were filtered out. Crucially, raw viral infection transcriptomes are dominated by pan-viral IFN signaling and innate immune responses. If used directly for CMap querying, this massive inflammatory signature biases the transcriptome signature reversion algorithm toward falsely prioritizing broad immunosuppressants (e.g., glucocorticoids) rather than identifying true host-directed antivirals, a confounding challenge in computational drug repurposing [18]. To abrogate this transcriptomic masking effect and isolate the viral manipulation of host metabolic programs, dominant IFN and generic antiviral-response genes were manually excluded from the upregulated gene set. Similarly, to prevent the spurious enrichment of highly toxic compounds, genes associated with non-specific cytotoxicity or global transcriptional shutdown were removed from the downregulated set. Following this curation, the top 150 upregulated and 150 downregulated genes for each virus were defined as the final dual-infection signatures (Figure 1). The explicitly excluded gene lists, final gene signatures, and applied statistical thresholds are fully detailed in Supplementary Table S2. Regarding the preparation of Figure 1, a generative artificial intelligence tool, ChatGPT Images 2.5 (OpenAI, San Francisco, CA, USA), was used to assist with the overall layout, schematic illustration, icon generation, and visual representation of the workflow. The generative AI tool was exclusively used for graphical representation and was not involved in the generation, modification, or interpretation of any experimental data, statistical analyses, bioinformatics calculations, or candidate drug selection. All scientific content, specific values, and methodological parameters depicted in Figure 1 were strictly determined and verified by the authors.

### 4.2. CLUE/CMap Analysis and Cross-Virus Compound Ranking

The curated DENV and ZIKV gene signatures were independently queried against the L1000-based CLUE CMap platform (Broad Institute, Cambridge, MA, USA). The resulting perturbagen profiles were cross-referenced with the DrugCentral database to filter for U.S. Food and Drug Administration (FDA)-, European Medicines Agency (EMA)-, and Pharmaceuticals and Medical Devices Agency (PMDA)-approved drugs, yielding 1921 matched compounds [43,44]. The specific CLUE query parameters (platform version, cell line, treatment duration, and score metric), the normalization scheme for the combined connectivity score, and the complete ranked list of 1921 compounds with per-virus scores are provided in Supplementary Table S2. To prioritize candidate interventions, a combined connectivity score was mathematically defined as the sum of the DENV and ZIKV connectivity scores. This consensus-scoring approach mitigates dataset-specific stochastic noise and constitutes an established computational strategy within the CMap framework to identify perturbagens that universally oppose overlapping disease states [16]. Compounds exhibiting the most negative combined scores—indicative of a strong transcriptional opposition to the viral infection state—were prioritized, and the top 100 candidates were advanced to the subsequent biological curation step.

### 4.3. Biological Curation and Candidate Selection

The top 100 in silico candidates underwent objective manual curation based on predefined criteria, in alignment with established expert triage guidelines for drug repurposing [14]: (1) exclusion of extensively documented direct-acting flavivirus inhibitors to maximize repurposing novelty; (2) diversity of mechanisms of action (MOA), particularly favoring interventions targeting host lipid metabolism and immunomodulation; and (3) experimental feasibility and commercial availability. For instance, clofibrate was selected purely based on its objective high ranking in the in silico CMap analysis to serve as a representative PPARα-modulating pharmacological probe, acknowledging its currently limited clinical use. This systematic triaging is a necessity in computational drug repurposing to eliminate redundant pharmacological classes and prioritize candidates with realistic translational potential [14]. This systematic triage narrowed the list to 70 prioritized compounds. Following procurement and availability review, 68 compounds were retained. After final exclusion of one compound, 67 structurally and pharmacologically diverse compounds were selected for in vitro phenotypic screening (Figure 1 and Supplementary Table S1).

### 4.4. Cell Culture and Viruses

African green monkey kidney epithelial cells (Vero E6; ATCC CRL-1586) were obtained from the American Type Culture Collection (ATCC, Manassas, VA, USA). Cells were cultured in Dulbecco’s Modified Eagle Medium (DMEM) supplemented with 10% fetal bovine serum (Thermo Fisher Scientific, Waltham, MA, USA) and 1% penicillin–streptomycin. Cultures were maintained at 37 °C in a humidified 5% CO_2_ atmosphere.

To ensure reproducibility and rule out reporter-specific artifacts, two distinct ZIKV models were used. For high-throughput screening, an infectious cDNA clone, pBAC-Venus-ZIKV-Paraiba, which is based on a previously described infectious clone system, was used to rescue a recombinant ZIKV expressing the Venus fluorescent protein (rZIKV-Venus) [45,46]. In this construct, the Venus reporter gene was integrated upstream of the precursor membrane (prM) region. The plasmid sequences were verified (Cosmogenetech Inc., Seoul, Republic of Korea). rZIKV-Venus was rescued via transfection of the plasmid into Vero E6 cells and subsequently propagated. Passage 4 virus stocks were used for all screening and validation assays and maintained at −80 °C. The infectious titer of the rZIKV-Venus working stock was quantified using a fluorescence focus-based titration assay. Vero E6 cells seeded in 96-well plates (1.0 × 10^4^ cells/well) were infected with 10-fold serial dilutions of the virus. At 72 h post-infection, Venus-positive fluorescent foci were enumerated via fluorescence microscopy. The viral titer was expressed as focus-forming units per milliliter (FFU/mL), with the specific stock used herein yielding a titer of 2.0 × 10^5^ FFU/mL.

For mechanistic validation against an authentic clinical isolate, the WT ZIKV strain PRVABC59 (Asian/American lineage), obtained from the National Culture Collection for Pathogens (Cheongju, Republic of Korea), was used. Both viruses were amplified and assayed in Vero E6 cells.

### 4.5. Antiviral Screening and 1% DMSO Normalization

Vero E6 cells were seeded in 96-well plates at a density of 1.0 × 10^4^ cells per well and incubated overnight. Cells were infected with rZIKV-Venus at 250 FFU per well (nominal multiplicity of infection (MOI) of 0.025). After 1 h of viral adsorption, the inoculum was replaced with fresh medium containing serially diluted candidate compounds. To eliminate solvent-induced experimental artifacts and ensure identical basal profiles across all test groups, candidate compounds were prepared from 10 mM DMSO stocks, and the final concentration of DMSO was normalized to exactly 1.0% (*v*/*v*) across all drug dilutions and vehicle-treated control wells. Chloroquine was included as a reference antiviral compound. The candidate library was supplied as 10 mM DMSO stocks and was therefore capped at 100 μM under the 1.0% (*v*/*v*) DMSO constraint. Chloroquine diphosphate (CAS 50-63-5; M.W. 515.9; Sigma-Aldrich C6628) is freely water-soluble and was instead dissolved in water as a 100 mM aqueous stock, filter-sterilised through a 0.22 μm membrane, protected from light, and diluted directly into culture medium. Because this vehicle was aqueous, the tested range could be extended to 1000 μM without exceeding the solvent constraint applied to the library; DMSO was added separately to the chloroquine dilutions and to their matched control wells so that the final DMSO concentration was 1.0% (*v*/*v*) in every well on the plate. Candidate compounds were tested as ten two-fold serial dilutions spanning 0.195–100 μM, and chloroquine over the corresponding ten-fold higher range of 1.95–1000 μM.

### 4.6. Cell Viability and Cytotoxicity Assessment (MTT Assay)

Compound-induced cytotoxicity was quantitatively evaluated using the MTT reduction assay to measure metabolic viability, superseding initial nuclear count-based estimations [47]. Uninfected Vero E6 cells (1.0 × 10^4^ cells/well) were treated with serially diluted compounds under identical temporal parameters (120 h continuous exposure, including a 1 h mock-adsorption step) and the strict 1% DMSO final concentration used for the antiviral screening assay. Following the incubation period, the medium was replaced with MTT solution, and the resulting intracellular formazan crystals were solubilized. The optical density was quantitatively measured at 540 nm using a Varioskan LUX multimode microplate reader (Thermo Fisher Scientific). Cell viability was calculated as a percentage relative to the 1% DMSO vehicle-treated control. The CC_50_ and IC_50_ were extrapolated via non-linear regression analysis using GraphPad Prism software (version 10.5.0; GraphPad Software, Boston, MA, USA). The SI was defined as the ratio of CC_50_ to IC_50_. To verify that the metabolic-reduction readouts were not artifactually skewed, direct cell survival was orthogonally confirmed by analyzing DAPI-positive nuclei counts from the 120 h fluorescence images. This confirmation was performed at the single working concentrations used in the mechanistic assays and does not constitute an independent, cell-count-derived CC_50_; all CC_50_ values and selectivity indices reported in this study were obtained from the MTT assay.

### 4.7. Combination Treatment and FICI Analysis

Drug–drug interactions between selected statins and clofibrate were mathematically assessed using the FICI. Vero E6 cells were infected with rZIKV-Venus and co-treated with fixed concentration ratios of statin–clofibrate combinations (4:1, 2:1, 1:1, 1:2, and 1:4, expressed throughout as clofibrate-to-statin) for 120 h. IC_50_ values were fitted separately for each of *n* = 3 independent biological replicates. To clarify the quantification, the FICI was calculated based on the individual concentration of each agent present within the mixture at the synergistic IC_50_ point, using the following explicit formula:

$$FICI\;=\frac{IC_{50}\;of\;Drug\;A\;in\;combination}{IC_{50}\;of\;Drug\;A\;alone}+\frac{IC_{50}\;of\;Drug\;B\;in\;combination}{IC_{50\;}of\;Drug\;B\;alone}$$

Crucially, the numerator represents the specific concentration of the individual drug within the combined mixture that achieves 50% viral inhibition, not the aggregate total concentration. The resulting FICI values were interpreted according to established pharmacological criteria: FICI ≤ 0.5 was defined as synergistic, 0.5 < FICI ≤ 4.0 as no interaction (additive or indifferent), and FICI > 4.0 as antagonistic [48]. The calculated FICI values across all tested concentration ratios (4:1, 2:1, 1:1, 1:2, and 1:4, clofibrate-to-statin), with replicate-level values and 95% confidence intervals, are provided in Supplementary Table S3. Among the evaluated ratios, the 4:1 combination of clofibrate to rosuvastatin demonstrated the optimal balance of synergistic efficacy and maximal selectivity (SI = 61.33) and was therefore prioritized for downstream mechanistic assays. Orthogonal response-surface synergy analysis was performed using the SynergyFinder Plus web application (version 3.0) applying the Bliss independence and ZIP models to generate 3D synergy landscapes and verify the interaction confidence.

### 4.8. OA-Mediated Metabolic Complementation and Fluorescence Analysis

To determine whether the antiviral effects of the rosuvastatin-clofibrate combination were mechanistically linked to host lipid depletion, an OA-mediated metabolic complementation assay was performed. Vero E6 cells were seeded in 8-well chamber slides at a density of 1.0 × 10^4^ cells/well and incubated overnight. To validate the phenotypes across different viral systems, cells were infected with either the reporter virus rZIKV-Venus or the authentic WT ZIKV strain (PRVABC59) at 250 FFU/well.

After 1 h of viral adsorption, the inoculum was replaced with fresh medium containing 1% DMSO vehicle, OA alone (100 μM), rosuvastatin (0.78 μM) plus clofibrate (3.12 μM) representing the optimized 4:1 synergistic ratio, or the triple combination (rosuvastatin, clofibrate, and OA) added simultaneously at 1 h post-infection. Experiments were performed in triplicate wells for each treatment group. At 120 h post-infection, cells were fixed with 4% paraformaldehyde for 10 min at room temperature.

The subsequent visualization strategy diverged based on the viral model used:

(1) For the rZIKV-Venus model, which inherently expresses the Venus fluorescent reporter, no exogenous antibody staining was required.
(2) For the non-reporter WT ZIKV model, fixed cells were permeabilized, blocked with 5% bovine serum albumin, and incubated overnight at 4 °C with the pan-flavivirus group envelope monoclonal antibody 4G2 (Native Antigen Company, Oxfordshire, UK). Following repeated washes, cells were incubated for 1 h at room temperature with an Alexa Fluor 488-conjugated goat anti-mouse IgG secondary antibody (Thermo Fisher Scientific, Waltham, MA, USA).

Following viral antigen detection, all samples underwent identical lipid and nuclear staining. Intracellular neutral LDs were stained with HCS LipidTOX Deep Red Neutral Lipid Stain (Thermo Fisher Scientific) according to the manufacturer’s instructions. Nuclei were counterstained with DAPI. Fluorescence images were acquired using an LSM 900 confocal microscope and analyzed via Zen Blue Software (version 3.1; Carl Zeiss, Jena, Germany) under identical acquisition settings across all experimental groups. Viral fluorescence signals, LipidTOX fluorescence intensity, and DAPI-positive nuclei were quantified using ImageJ/Fiji software (version 1.53c; National Institutes of Health, Bethesda, MD, USA) [49]. Viral fluorescence intensity and LipidTOX fluorescence intensity were normalized to the respective cell numbers to account for variations in cell density.

### 4.9. Viral RNA Extraction and RT-qPCR

To quantify intracellular viral genome replication independently of extracellular release kinetics, the culture media were completely discarded at 120 h post-infection, and the remaining cell pellets were washed. Total intracellular RNA was extracted directly from the cell pellets using the QIAamp Viral RNA Mini Kit (QIAGEN, Venlo, The Netherlands). ZIKV RNA levels were quantified using the TOPreal One-step RT-qPCR Kit (SYBR Green with low ROX; Enzynomics, Daejeon, Republic of Korea). The ZIKV-specific primers used were: MF944 (forward), 5′-GGTCATGATACTGCTGATTGC-3′; and ER1269 (reverse), 5′-CCACTAACGTTCTTTTGCAGAC-3′. To normalize viral RNA copies against variations in viable cell numbers and RNA extraction efficiency, the host housekeeping gene β-actin (ACTB) was amplified in parallel as an endogenous internal control. Relative intracellular ZIKV RNA levels were calculated using the 2^−∆∆C^_T_ method [50] and expressed as fold-changes relative to the vehicle-treated infected control.

### 4.10. Infectious Virus Titration (TCID_50_ Assay)

To quantify the total production of functional progeny virions, encompassing both released and intracellularly retained particles, a modified TCID_50_ assay was performed. Because a significant proportion of mature flavivirus virions remains sequestered within the host cell endomembrane system prior to exocytosis, the entire contents of the wells—comprising both the cell monolayer and the culture supernatant—were subjected to three consecutive freeze–thaw cycles (−80 °C to 37 °C) at 120 h post-infection. This mechanical disruption ensures the complete release of intracellularly assembled virions without compromising viral infectivity [11]. The resulting crude lysates were clarified by centrifugation to remove cellular debris. The clarified homogenates were serially diluted ten-fold in unsupplemented DMEM and inoculated onto naive, confluent Vero E6 monolayers in 96-well plates. Following a 4-day incubation at 37 °C under 5% CO_2_, virus-induced cytopathic effects (CPE) were microscopically evaluated. Infectious viral titers were calculated using the Reed–Muench method and expressed as log_10_ TCID_50_/mL.

### 4.11. Statistical Analysis

Statistical analyses were performed using GraphPad Prism software (version 10.5.0). All quantitative data are derived from independent biological replicates generated from separate virus infections and drug treatments performed on different days. Replicate numbers were *n* = 3 for the monotherapy dose–response screening (Figure 2, Table 1) and for the combination experiments (Figure 3, Supplementary Table S3 and Figure S1), and *n* = 4 for the metabolic complementation assays (Figure 4 and Figure 5) and the viability validation (Supplementary Figure S2); each figure and table legend states its own replicate number.

The specific statistical methodologies applied were as follows:

(1) For the IC_50_ and CC_50_ concentrations from dose–response assays, non-linear regression analysis (four-parameter logistic model) was used.
(2) For multi-group comparisons across independent experimental conditions (e.g., comparing relative fluorescence intensities, LipidTOX fluorescence intensity, intracellular viral RNA copies, and TCID_50_ infectious titers among Vehicle, ROS + CLO, OA, and ROS + CLO + OA groups), statistical significance was determined using ordinary one-way analysis of variance (ANOVA). To control the family-wise error rate across all pairwise comparisons, Tukey’s multiple-comparison post hoc test was applied.
(3) Pearson correlation analysis was performed to evaluate the relationship between host LD availability and viral replication readouts. Each point in this analysis is one independent biological replicate, and the coefficient is computed across the four treatment groups pooled. Because the groups differ by design in both variables, the pooled coefficient reflects between-group separation as well as any within-group association and is reported as a measure of concordance between treatment conditions, not as evidence of a graded within-group dose–response relationship. Within-group coefficients were not computed as hypothesis tests, because four replicates per group provide two degrees of freedom and therefore no usable power.

*p*-values < 0.05 were considered statistically significant and are denoted by asterisks in the figures (* *p* < 0.05, ** *p* < 0.01, *** *p* < 0.001, **** *p* < 0.0001; ns, not significant).

## 5. Conclusions

This study demonstrates that the combination of atorvastatin or rosuvastatin with clofibrate exerts highly synergistic antiviral effects against ZIKV through concurrent pharmacological modulation of host lipid metabolism. Crucially, OA-mediated metabolic complementation, validated across both reporter and authentic WT ZIKV models, provided biological evidence that viral genome replication and infectious progeny production are highly dependent on the availability of host LDs. These findings provide robust experimental support for targeting lipid metabolic pathways as a host-directed intervention and demonstrate the utility of highly curated transcriptomic signature-based drug repurposing as a mechanistic prioritization strategy for antiviral candidate discovery.

## Figures and Tables

**Table 1:** In vitro antiviral efficacies and cytotoxicity profiles of the 67 candidate compounds.

No.	Compound	CC_50_ (µM)	IC_50_ (µM)	SI
1	Levomepromazine	>100	>100	N.C.
2	Vecuronium bromide	85.15	84.31	1.00
3	Atorvastatin	27.35	2.29	11.94
4	Palonosetron hydrochloride	>100	7.91	>12.64
5	Imiquimod	>100	>100	N.C.
6	Valproic acid	22.80	38.02	0.59
7	Lenalidomide	>100	99.28	>1.00
8	Quinine	>100	59.02	>1.69
9	Docetaxel	0.42	0.38	1.10
10	Temozolomide	>100	>100	N.C.
11	Tofacitinib	>100	>100	N.C.
12	Maraviroc	>100	>100	N.C.
13	Sodium 2-mercaptoethanesulfonate	>100	>100	N.C.
14	Phensuximide	>100	>100	N.C.
15	Retinyl acetate	60.12	44.16	1.36
16	Guaifenesin	>100	>100	N.C.
17	Carprofen	94.18	>100	<0.94
18	Sitagliptin	>100	>100	N.C.
19	Rivaroxaban	>100	>100	N.C.
20	Fostamatinib	8.04	8.32	0.96
21	Naltrexone	>100	>100	N.C.
22	Mupirocin	>100	>100	N.C.
23	Deferiprone	>100	>100	N.C.
24	Febuxostat	>100	>100	N.C.
25	Toremifene	16.03	12.53	1.27
26	Doxercalciferol	8.43	53.21	0.15
27	Maprotiline hydrochloride	19.86	36.06	0.55
28	Sulfacetamide	92.08	>100	<0.92
29	Brinzolamide	>100	>100	N.C.
30	Mecillinam	>100	>100	N.C.
31	Pargyline	>100	>100	N.C.
32	Varenicline	>100	62.38	>1.60
33	Cinacalcet	29.31	37.41	0.78
34	Bosutinib	0.68	1.09	0.62
35	Pioglitazone	>100	76.92	>1.30
36	Ruxolitinib	89.96	>100	<0.89
37	Safinamide	>100	56.50	>1.76
38	Doxylamine succinate	>100	>100	N.C.
39	Cefoxitin	>100	>100	N.C.
40	Naftifine hydrochloride	82.80	26.98	3.06
41	Scopolamine butylbromide	>100	79.44	>1.25
42	Tegafur	>100	>100	N.C.
43	Entacapone	31.05	29.04	1.06
44	Propoxycaine hydrochloride	>100	44.57	>2.24
45	D-Cycloserine	>100	>100	N.C.
46	Levocetirizine	>100	98.14	>1.01
47	Nifurtimox	98.48	88.46	1.11
48	Didanosine	>100	70.15	>1.42
49	Acetohydroxamic acid	>100	6.98	>14.32
50	Guanabenz	63.25	43.55	1.45
51	Rosuvastatin	61.52	5.68	10.83
52	Rifaximin	89.75	55.85	1.60
53	Flumazenil	>100	31.77	>3.14
54	Paclitaxel	0.71	0.95	0.74
55	(+)-Tubocurarine chloride pentahydrate	>100	99.48	>1.00
56	Chlortetracycline hydrochloride	>100	>100	N.C.
57	Spectinomycin sulfate	89.90	80.45	1.11
58	Meclofenamic acid sodium salt	84.84	79.60	1.06
59	10,11-Dihydrocarbamazepine	60.19	52.78	1.14
60	Clofibrate	98.50	20.75	4.74
61	(±)-Verapamil hydrochloride	58.17	49.89	1.16
62	Pantoprazole Sulfide	25.47	49.83	0.51
63	Phenytoin Sodium	>100	89.38	>1.11
64	Hyodeoxycholic Acid	>100	91.92	>1.08
65	Metoclopramide	92.45	89.64	1.03
66	Prochlorperazine Dimaleate	45.31	60.20	0.75
67	Dibenzocyclooctyne-maleimide	53.01	42.31	1.25

CC_50_, half-maximal cytotoxic concentration; IC_50_, half-maximal inhibitory concentration; SI, selectivity index (calculated as CC_50_/IC_50_). N.C., not calculable—both CC_50_ and IC_50_ exceeded the maximum tested concentration, so no bound can be placed on their ratio. Compounds were tested as ten two-fold serial dilutions spanning 0.195–100 µM. CC_50_ and IC_50_ were estimated by four-parameter logistic non-linear regression within this range; values that could not be resolved at or below 100 µM are reported as “>100” rather than as regression extrapolations beyond the tested range. For SI, “>” denotes a lower bound (censored CC_50_ with finite IC_50_, calculated as 100/IC_50_) and “<” denotes an upper bound (finite CC_50_ with censored IC_50_, calculated as CC_50_/100). All SI values, bounds and point estimates alike, were truncated to two decimal places.

**Table 2:** Comparison of the in vitro anti-ZIKV potency of rosuvastatin and atorvastatin with their clinically achievable total and unbound plasma exposure.

Parameter	Rosuvastatin	Atorvastatin
In vitro IC_50_ against rZIKV-Venus (µM)	5.68	2.29
Maximum approved daily dose	40 mg	80 mg
Total C_m_ₐ_x_ (µM)	0.048	0.151
Plasma protein binding (%)	88	≥98
Unbound C_m_ₐ_x_ (µM)	0.0058	0.0030
IC_50_/unbound C_m_ₐ_x_	≈1000	≈760

IC_50_ values are those determined in the present study against rZIKV-Venus in Vero E6 cells (Table 1, Figure 2). Total C_m_ₐ_x_ was converted to molar units using the free-acid molecular weights (rosuvastatin, 481.54 g/mol; atorvastatin, 558.64 g/mol), which correspond both to the compound forms tested here (CAS 287714-41-4 and 134523-00-5) and to the species quantified in the cited pharmacokinetic studies. For atorvastatin, C_m_ₐ_x_ is the measured value after a single 80 mg oral dose in healthy adults (84.3 ± 39.4 ng/mL) [37]. For rosuvastatin, no directly measured C_m_ₐ_x_ at the maximum approved 40 mg dose is available; the value used (23.2 ng/mL) was obtained from the 10 mg single-dose value of 5.8 ng/mL by dose proportionality, which the product information states explicitly for both C_m_ₐ_x_ and AUC [38]. Plasma protein binding was taken from the respective prescribing information [38,39], and unbound C_m_ₐ_x_ is the product of total C_m_ₐ_x_ and the unbound fraction implied by that binding. Reported C_m_ₐ_x_ values carry substantial between-subject variability, so the ratios in the final row are given to one significant figure. Clofibrate is not included in this comparison because it is a prodrug whose conversion to clofibric acid was not verified in this cell model, so clinical exposure data describing circulating clofibric acid cannot be compared with the in vitro potency of the applied prodrug (see Section 3). Abbreviations: C_m_ₐ_x_, maximal plasma concentration; IC_50_, half-maximal inhibitory concentration; ZIKV, Zika virus.

## Data Availability

The public dengue virus (DENV) and Zika virus (ZIKV) infection transcriptomic datasets analyzed in this study are openly available from the NCBI Gene Expression Omnibus (GEO) under accession numbers GSE84285 and GSE93385, respectively. Drug annotation information was obtained from the DrugCentral database, and transcriptional connectivity analyses were performed using the CLUE Connectivity Map platform. All other data supporting the findings of this study are included within the article and its Supplementary Materials.

## Supplementary Materials

The following supporting information can be downloaded at: https://www.mdpi.com/article/10.3390/ijms27219637/s1.

## Abbreviations

The following abbreviations are used in this manuscript:

ZIKV	Zika virus
DENV	Dengue virus
rZIKV-Venus	Recombinant Zika virus expressing Venus fluorescent protein
CMap	Connectivity Map
CLUE	Connectivity Map Linked User Environment
IFN	Interferon
ER	Endoplasmic reticulum
LD	Lipid droplet
OA	Oleic acid
WT	Wild-type
HMG-CoA	3-Hydroxy-3-methylglutaryl-coenzyme A
PPARα	Peroxisome proliferator-activated receptor alpha
SREBP2	Sterol regulatory element-binding protein 2
GGPP	Geranylgeranyl pyrophosphate
FPP	Farnesyl pyrophosphate
DGAT	Diacylglycerol acyltransferase
CC_50_	Half-maximal cytotoxic concentration
IC_50_	Half-maximal inhibitory concentration
SI	Selectivity index
FICI	Fractional inhibitory concentration index
MOI	Multiplicity of infection
DMSO	Dimethyl sulfoxide
MTT	3-(4,5-Dimethylthiazol-2-yl)-2,5-diphenyltetrazolium bromide
DAPI	4′,6-Diamidino-2-phenylindole
RT-qPCR	Reverse transcription quantitative polymerase chain reaction
TCID_50_	50% tissue culture infectious dose
ANOVA	Analysis of variance
SD	Standard deviation
ATO	Atorvastatin
ROS	Rosuvastatin
CLO	Clofibrate
GEO	Gene Expression Omnibus
NCBI	National Center for Biotechnology Information
FDA	U.S. Food and Drug Administration
EMA	European Medicines Agency
PMDA	Pharmaceuticals and Medical Devices Agency
ATCC	American Type Culture Collection
DMEM	Dulbecco’s modified Eagle’s medium
FBS	Fetal bovine serum
FFU	Focus-forming unit
EBOV	Ebola virus
