# Supplementary material for: Synergistic Anti-Zika Activity of Repurposed Statin and Clofibrate Combinations: Evidence from Oleic Acid-Mediated Metabolic Complementation
Source: Int J Mol Sci. 2026 Oct 28;27(21):9637. doi: 10.3390/ijms27219637 (PMC13763463; doi:10.3390/ijms27219637)
Supplement: Supplementary file 1 [file ijms-27-09637-s001.zip › Supplementary Materials.pdf]

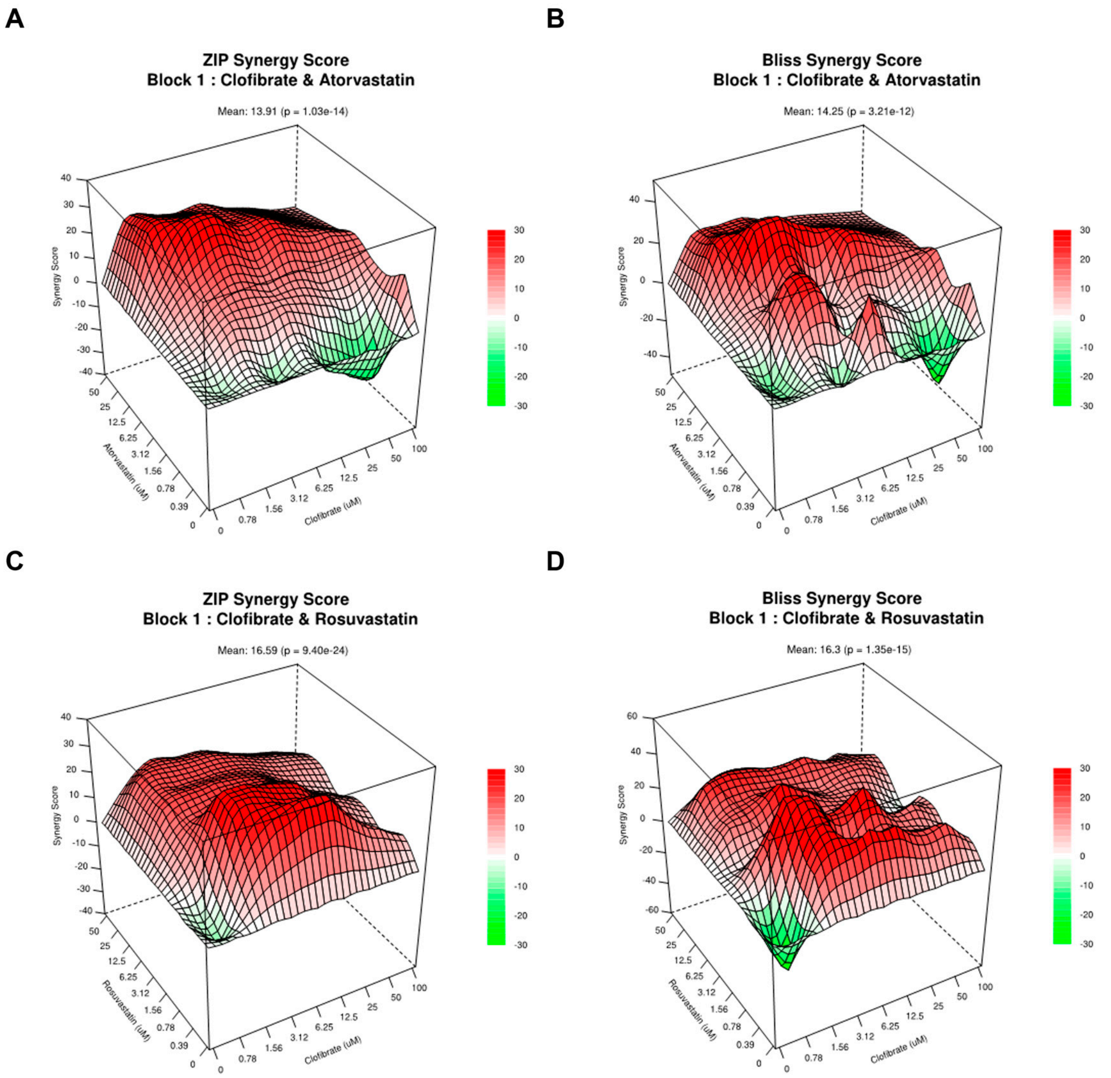

Supplementary Figure S1. Orthogonal validation of statin–clofibrate synergy via 3D response-surface modeling. The dose-response matrix data for (A, B) atorvastatin (ATO) + clofibrate (CLO) and (C, D) rosuvastatin (ROS) + clofibrate (CLO) combinations were analyzed using the SynergyFinder web application (version 3.0). The 3D synergy landscapes were generated using the Zero Interaction Potency (ZIP) model (A, C) and Bliss independence model (B, D). The landscapes shown were generated from the mean response matrix of three independent biological experiments, and the mean synergy score of each landscape is printed on the corresponding panel. Each replicate response matrix was additionally analyzed separately, and the per-replicate scores with the resulting mean and its 95% confidence interval are: ATO + CLO, ZIP — 14.01, 14.35, 13.38; mean 13.91 (95% CI 12.69–15.14); ATO + CLO, Bliss — 14.33, 14.55, 13.86; mean 14.25 (95% CI 13.37–15.12); ROS + CLO, ZIP — 17.20, 16.72, 15.85; mean 16.59 (95% CI 14.89–18.29); ROS + CLO, Bliss — 16.85, 16.42, 15.64; mean 16.30 (95% CI 14.78–17.83). Confidence intervals were obtained as mean ± $t_{0.975,2} \times \mathrm{SD}/\sqrt{3}$ ($t = 4.303$, $n = 3$) and therefore describe between-experiment reproducibility. The entire interval lies above zero for all four analyses. These positive synergy scores consistently support synergistic interactions between the statins and clofibrate. Abbreviations: ATO, atorvastatin; Bliss, Bliss independence model; CI, confidence interval; CLO, clofibrate; ROS, rosuvastatin; SD, standard deviation; ZIP, Zero Interaction Potency model.

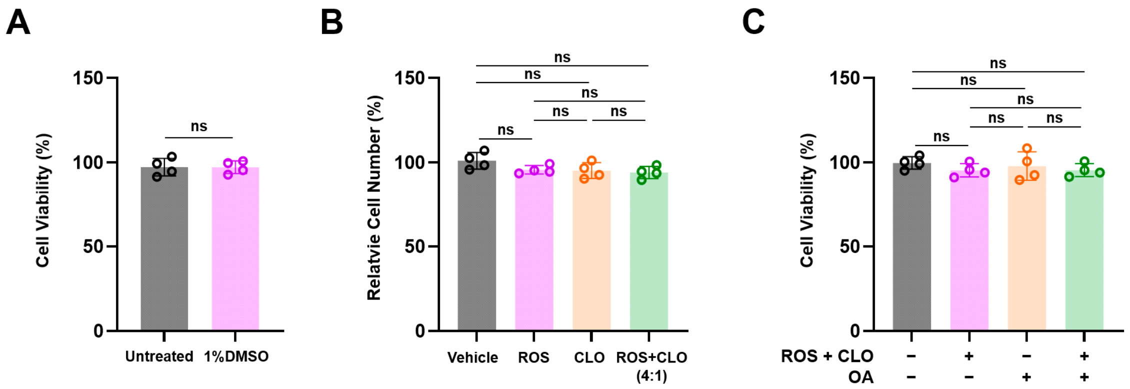

Supplementary Figure S2. Orthogonal validation of cell viability and solvent safety. (**A**) MTT-based cell viability of untreated cells and cells exposed to 1% DMSO for 120 h. (**B**) Direct cell counting based on DAPI-positive nuclei after 120 h of treatment with vehicle (1% DMSO), rosuvastatin alone (ROS, 0.78 μM), clofibrate alone (CLO, 3.12 μM), or the ROS + CLO combination at the 4:1 clofibrate-to-rosuvastatin ratio (0.78 μM + 3.12 μM. Cell numbers were normalized to the vehicle control. These are the single working concentrations used in the mechanistic complementation assays; no concentration series was tested in this experiment, so these data confirm that cell number is preserved at the working doses but do not constitute an independent, cell-count-derived CC_{50}. All CC_{50} values and selectivity indices reported in this study, including SI = 61.33, are MTT-derived. Data are from n = 4 independent biological replicates.

Supplementary Table S1. Regulatory and pharmacological classification of the 67 compounds selected for screening

No.	Compound	CAS No.	Entity type	FDA	EMA	PMDA	Pharmacological category
1	Levomepromazine	60-99-1	P.-D.A.C.	N.I.	N.I.	P.O.	CNS Agents (Neuro/Psych)
2	Vecuronium bromide	50700-72-6	A.S.F.	A	N.I.	A	Muscle Relaxants
3	Atorvastatin	134523-00-5	P.-D.A.C.	P.O.	N.I.	P.O.	Cardiovascular & Lipid-regulating
4	Palonosetron hydrochloride	135729-62-3	A.S.F.	A	A	A	Gastrointestinal Agents
5	Imiquimod	99011-02-6	A.A.I.	A	A	A	Anti-infectives (Antibiotics/Antivirals)
6	Valproic acid	99-66-1	A.A.I.	A	N.I.	A	CNS Agents (Neuro/Psych)
7	Lenalidomide	191732-72-6	A.A.I.	A	A	A	Antineoplastics & Immunomodulators
8	Quinine	130-95-0	P.-D.A.C.	P.O.	N.I.	P.O.	Anti-infectives (Antibiotics/Antivirals)
9	Docetaxel	114977-28-5	A.A.I.	A	A	A	Antineoplastics & Immunomodulators
10	Temozolomide	85622-93-1	A.A.I.	A	A	A	Antineoplastics & Immunomodulators
11	Tofacitinib	477600-75-2	P.-D.A.C.	P.O.	P.O.	P.O.	Antineoplastics & Immunomodulators
12	Maraviroc	376348-65-1	A.A.I.	A	A	A	Anti-infectives (Antibiotics/Antivirals)
13	Sodium 2-mercaptoethanesulfonate	19767-45-4	A.S.F.	A	N.I.	A	Others (Antidotes, Vitamins, Misc)
14	Phensuximide	86-34-0	A.A.I.	A	N.I.	N.I.	CNS Agents (Neuro/Psych)
15	Retinyl acetate	127-47-9	A.A.I.	N.I.	N.I.	A	Others (Antidotes, Vitamins, Misc)
16	Guaifenesin	93-14-1	A.A.I.	A	N.I.	A	Respiratory & Anti-allergy
17	Carprofen	53716-49-7	A.A.I.	A	N.I.	N.I.	NSAIDs & Analgesics
18	Sitagliptin	486460-32-6	P.-D.A.C.	P.O.	P.O.	P.O.	Metabolic & Endocrine
19	Rivaroxaban	366789-02-8	A.A.I.	A	A	A	Cardiovascular & Lipid-regulating
20	Fostamatinib	901119-35-5	P.-D.A.C.	P.O.	P.O.	P.O.	Antineoplastics & Immunomodulators
21	Naltrexone	16590-41-3	P.-D.A.C.	P.O.	P.O.	N.I.	CNS Agents (Neuro/Psych)
22	Mupirocin	12650-69-0	A.A.I.	A	N.I.	P.O.	Anti-infectives (Antibiotics/Antivirals)
23	Deferiprone	30652-11-0	A.A.I.	A	A	N.I.	Others (Antidotes, Vitamins, Misc)
24	Febuxostat	144060-53-7	A.A.I.	A	A	A	Others (Antidotes, Vitamins, Misc)

No.	Compound	CAS No.	Entity type	FDA	EMA	PMDA	Pharmacological category
25	Toremifene	89778-26-7	P.-D.A.C.	P.O.	P.O.	P.O.	Antineoplastics & Immunomodulators
26	Doxercalciferol	54573-75-0	A.A.I.	A	N.I.	N.I.	Others (Antidotes, Vitamins, Misc)
27	Maprotiline hydrochloride	10347-81-6	A.S.F.	A	N.I.	A	CNS Agents (Neuro/Psych)
28	Sulfacetamide	144-80-9	P.-D.A.C.	P.O.	N.I.	N.I.	Anti-infectives (Antibiotics/Antivirals)
29	Brinzolamide	138890-62-7	A.A.I.	A	A	A	Others (Antidotes, Vitamins, Misc)
30	Mecillinam	32887-01-7	P.-D.A.C.	P.O.	N.I.	N.I.	Anti-infectives (Antibiotics/Antivirals)
31	Pargyline	555-57-7	P.-D.A.C.	P.O.	N.I.	N.I.	CNS Agents (Neuro/Psych)
32	Varenicline	249296-44-4	P.-D.A.C.	P.O.	P.O.	P.O.	CNS Agents (Neuro/Psych)
33	Cinacalcet	226256-56-0	P.-D.A.C.	P.O.	P.O.	P.O.	Metabolic & Endocrine
34	Bosutinib	380843-75-4	P.-D.A.C.	P.O.	P.O.	P.O.	Antineoplastics & Immunomodulators
35	Pioglitazone	111025-46-8	P.-D.A.C.	P.O.	P.O.	P.O.	Metabolic & Endocrine
36	Ruxolitinib	941678-49-5	P.-D.A.C.	P.O.	P.O.	P.O.	Antineoplastics & Immunomodulators
37	Safinamide	133865-89-1	P.-D.A.C.	P.O.	P.O.	P.O.	CNS Agents (Neuro/Psych)
38	Doxylamine succinate	562-10-7	A.S.F.	A	N.I.	N.I.	Respiratory & Anti-allergy
39	Cefoxitin	35607-66-0	P.-D.A.C.	P.O.	N.I.	N.I.	Anti-infectives (Antibiotics/Antivirals)
40	Naftifine hydrochloride	65473-14-5	A.S.F.	A	N.I.	N.I.	Anti-infectives (Antibiotics/Antivirals)
41	Scopolamine butylbromide	149-64-4	A.S.F.	N.I.	N.I.	A	Gastrointestinal Agents
42	Tegafur	17902-23-7	A.A.I.	N.I.	A	A	Antineoplastics & Immunomodulators
43	Entacapone	130929-57-6	A.A.I.	A	A	A	CNS Agents (Neuro/Psych)
44	Propoxycaine hydrochloride	550-83-4	A.S.F.	A	N.I.	N.I.	NSAIDs & Analgesics
45	D-Cycloserine	68-41-7	A.A.I.	A	N.I.	A	Anti-infectives (Antibiotics/Antivirals)
46	Levocetirizine	130018-77-8	P.-D.A.C.	P.O.	N.I.	P.O.	Respiratory & Anti-allergy
47	Nifurtimox	23256-30-6	A.A.I.	A	N.I.	N.I.	Anti-infectives (Antibiotics/Antivirals)
48	Didanosine	69655-05-6	A.A.I.	A	N.I.	A	Anti-infectives (Antibiotics/Antivirals)
49	Acetohydroxamic acid	546-88-3	A.A.I.	A	N.I.	N.I.	Others (Antidotes, Vitamins, Misc)

No.	Compound	CAS No.	Entity type	FDA	EMA	PMDA	Pharmacological category
50	Guanabenz	5051-62-7	P.-D.A.C.	P.O.	N.I.	P.O.	Cardiovascular & Lipid-regulating
51	Rosuvastatin	287714-41-4	P.-D.A.C.	P.O.	N.I.	P.O.	Cardiovascular & Lipid-regulating
52	Rifaximin	80621-81-4	A.A.I.	A	N.I.	A	Anti-infectives (Antibiotics/Antivirals)
53	Flumazenil	78755-81-4	A.A.I.	A	N.I.	A	CNS Agents (Neuro/Psych)
54	Paclitaxel	33069-62-4	A.A.I.	A	A	A	Antineoplastics & Immunomodulators
55	(+)-Tubocurarine chloride pentahydrate	6989-98-6	A.S.F.	A	N.I.	N.I.	Muscle Relaxants
56	Chlortetracycline hydrochloride	64-72-2	A.S.F.	A	N.I.	N.I.	Anti-infectives (Antibiotics/Antivirals)
57	Spectinomycin sulfate	23312-56-3	A.S.F.	A	N.I.	A	Anti-infectives (Antibiotics/Antivirals)
58	Meclofenamic acid sodium salt	6385-02-0	A.S.F.	A	N.I.	N.I.	NSAIDs & Analgesics
59	10,11-Dihydrocarbamazepine	3564-73-6	M./I.	P.O.	N.I.	P.O.	Others (Antidotes, Vitamins, Misc)
60	Clofibrate	637-07-0	A.A.I.	A	N.I.	A	Cardiovascular & Lipid-regulating
61	(±)-Verapamil hydrochloride	152-11-4	A.S.F.	A	N.I.	A	Cardiovascular & Lipid-regulating
62	Pantoprazole Sulfide	102625-64-9	M./I.	P.O.	P.O.	P.O.	Gastrointestinal Agents
63	Phenytoin Sodium	630-93-3	A.S.F.	A	N.I.	A	CNS Agents (Neuro/Psych)
64	Hyodeoxycholic Acid	83-49-8	R.R./O.	N.I.	N.I.	N.I.	Gastrointestinal Agents
65	Metoclopramide	364-62-5	P.-D.A.C.	P.O.	N.I.	A	Gastrointestinal Agents
66	Prochlorperazine Dimaleate	84-02-6	A.S.F.	A	N.I.	A	CNS Agents (Neuro/Psych)
67	Dibenzocyclooctyne-maleimide	1395786-30-7	R.R./O.	N.I.	N.I.	N.I.	Others (Antidotes, Vitamins, Misc)

[1] Entity Type: A.A.I., Approved active ingredient; A.S.F., Approved salt/form; P.-D.A.C., Parent-drug-associated compound; M./I., Metabolite/impurity; R.R./O., Research reagent/other.

[2] Regulatory Status (FDA/EMA/PMDA): A, Approved (indicating explicit approval of the listed entity/form); P.O., Parent only (indicating that while the specific listed form is not directly approved, an approved parent active ingredient or a closely related drug form was identified); N.I., Not identified.

[3] Note: EMA status strictly refers to centrally authorized medicines via EMA/EPAR; national or decentralized EU authorizations alone were not counted. Historical or off-market approvals were retained for comprehensive screening. Regulatory status was verified as of September 2026

Supplementary Table S2. Comprehensive computational parameters and datasets for transcriptome-guided drug repurposing (**Provided as a separate Excel file**). This supplementary dataset provides the complete parameters required for independent reproduction of the in silico screening pipeline. **Sheet 1** lists the final curated dual-virus gene expression signatures. **Sheet 2** details the explicitly excluded interferon-stimulated and nonspecific cytotoxicity genes removed to prevent transcriptomic masking. **Sheet 3** explicitly outlines the CLUE Connectivity Map query parameters and the normalization scheme for the combined connectivity score. **Sheet 4** provides the complete ranked list of all 1,921 DrugCentral-matched candidate compounds along with their individual and combined connectivity scores.

Supplementary Table S3. Ratio-specific fractional inhibitory concentration index (FICI) values for statin–clofibrate combinations against rZIKV-Venus.

Combination	CLO:statin ratio	IC_{50} statin alone (μM)	IC_{50} CLO alone (μM)	Statin in combination (μM)	CLO in combination (μM)	FICI rep 1	FICI rep 2	FICI rep 3	Mean FICI (95% CI)	Interpretation
ATO + CLO	4:1	2.29	20.75	0.322	1.288	0.196	0.178	0.234	0.203 (0.132–0.274)	Synergy
ATO + CLO	2:1	2.29	20.75	0.780	1.560	0.439	0.386	0.423	0.416 (0.348–0.484)	Synergy
ATO + CLO	1:1	2.29	20.75	0.895	0.895	0.433	0.430	0.440	0.434 (0.421–0.447)	Synergy
ATO + CLO	1:2	2.29	20.75	1.560	0.780	0.775	0.626	0.755	0.719 (0.518–0.920)	Not synergistic
ATO + CLO	1:4	2.29	20.75	1.893	0.473	0.864	0.966	0.718	0.849 (0.540–1.159)	Not synergistic
ROS + CLO	4:1	5.68	20.75	0.318	1.272	0.155	0.104	0.093	0.117 (0.034–0.200)	Synergy
ROS + CLO	2:1	5.68	20.75	1.560	3.120	0.362	0.449	0.464	0.425 (0.288–0.562)	Synergy
ROS + CLO	1:1	5.68	20.75	2.150	2.150	0.499	0.467	0.480	0.482 (0.442–0.522)	Synergy
ROS + CLO	1:2	5.68	20.75	2.726	1.363	0.457	0.513	0.667	0.546 (0.276–0.815)	Not synergistic
ROS + CLO	1:4	5.68	20.75	3.120	0.780	0.569	0.533	0.659	0.587 (0.425–0.749)	Not synergistic

Ratios are expressed as clofibrate-to-statin (CLO:statin), the orientation used throughout the main text. Vero E6 cells were infected with rZIKV-Venus and co-treated with fixed-ratio statin–clofibrate combinations for 120 h. FICI was calculated as (statin concentration in combination / statin IC_{50} alone) + (CLO concentration in combination / CLO IC_{50} alone), where each numerator is the concentration of that individual agent within the mixture at 50% viral inhibition, not the aggregate total. Single-agent and combination IC_{50} values were fitted separately for each of n = 3 independent biological replicates, performed from separate infections and drug treatments on different days. The mean FICI is the arithmetic mean of the three replicate values and the 95% confidence interval was obtained as mean ± $t_{0.975,2}$ × SD/√3 (t = 4.303). Interpretation follows the established convention applied to the mean FICI: ≤ 0.5, synergy; 0.5 < FICI ≤ 4.0, no interaction (additive or indifferent); > 4.0, antagonism. The 95% confidence interval lies entirely below 0.5 for the ATO + CLO 4:1, 2:1 and 1:1 combinations and for the ROS + CLO 4:1 combination, and entirely above 0.5 for the ATO + CLO 1:2 and 1:4 combinations; for the remaining four combinations the interval includes 0.5, so the interpretation given for those rows rests on the mean alone and should be read as provisional. Replicate-level values are reported so that every mean and interval in this table can be recomputed independently. Abbreviations: ATO, atorvastatin; CLO, clofibrate; ROS, rosuvastatin; FICI, fractional inhibitory concentration index; IC_{50}, half-maximal inhibitory concentration; SD, standard deviation; CI, confidence interval.
